# Hypoxia Regulates the Proliferation and Apoptosis of Coronary Artery Smooth Muscle Cells Through HIF-1α Mediated Autophagy in Yak

**DOI:** 10.3390/biom15020256

**Published:** 2025-02-10

**Authors:** Shanshan Yang, Yan Cui, Rui Ma, Sijiu Yu, Hui Zhang, Pengfei Zhao, Junfeng He

**Affiliations:** 1 Laboratory of Animal Anatomy & Tissue Embryology, Department of Basic Veterinary Medicine, Faculty of Veterinary Medicine, Gansu Agricultural University, Lanzhou 730070, China; 107332101038@st.gsau.edu.cn (S.Y.); 107332001032@st.gsau.edu.cn (R.M.); yusj@gsau.edu.cn (S.Y.); 107332101034@st.gsau.edu.cn (H.Z.); 107332201037@st.gsau.edu.cn (P.Z.); hejf@gsau.edu.cn (J.H.); 2 Gansu Province Livestock Embryo Engineering Research Center, Department of Clinical Veterinary Medicine, Faculty of Veterinary Medicine, Gansu Agricultural University, Lanzhou 730070, China

**Keywords:** heart coronary smooth muscle cells, migration, proliferation, hypoxia, autophagy, yak

## Abstract

Cell proliferation and migration mediated by hypoxia-inducible factor-1α (HIF-1α) are important processes of hypoxic cardiac vascular remodeling. HIF-1α also regulates the physiological hypoxic adaptation of the coronary artery in the yak heart, but the potential mechanism remains to be completely elucidated. In this study, coronary artery proliferation increased with age and hypoxia adaptation time. In vitro analysis showed that hypoxia can promote the proliferation of coronary vascular smooth muscle cells (CASMCs). Meanwhile, HIF-1α plays an important role in the regulation of proliferation and migration under hypoxia. Autophagy regulates cell proliferation and migration to participate in hypoxia adaptation in plateau animals. Here, the level of autophagy increased significantly in yak coronary arteries with age and was regulated by HIF-1α-mediated hypoxia. In addition, autophagy could also mediate the hypoxic effect on the proliferation and migration of CASMCs. In summary, the results revealed that the increase in yak heart coronary artery thickening with age increases vascular smooth muscle cell proliferation and migration, mainly achieved through hypoxia-mediated HIF-1α-regulated autophagy. These results contribute to understanding how the heart adapts to life in a hypoxic environment.

## 1. Introduction

The Tibetan Plateau, with an average altitude of greater than 4000 m, is characterized by hypoxia, low air pressure, and low temperature. Several studies have reported that the migration of ordinary plain animals to a hypoxic environment of the plateau can lead to symptoms including pulmonary hypertension and right heart hypertrophy [1,2]. *Yak*, as a representative species, lives in high-altitude areas for a long time but does not show plateau pathological symptoms in its heart [3]. The heart of a yak plays an important role in the process of adapting to hypoxia. The coronary artery, as a nutrient vessel of the heart, is more seriously affected by hemodynamic effects than the arteries of other organs. Its high sensitivity to changes in hypoxic environments makes it the focus of research on hypoxic vascular remodeling [4]. Hypoxia-induced proliferation, migration, and apoptosis of coronary artery media smooth muscle cells (CASMCs) can significantly cause intimal thickening, arterial stenosis, and vascular remodeling. This is also an important pathological feature of cardiovascular diseases such as coronary artery disease, hypertension, atherosclerosis, and heart failure [5]. Therefore, studying the hypoxia adaptability of yak coronary arteries and the associated specific molecular mechanisms is highly significant [6].

Cardiovascular cell proliferation and migration mediated by hypoxia-inducible factor-1α (HIF-1α) is a typical reconstruction process [7]. HIF-1α is a core regulator of angiogenesis under hypoxic conditions. Under normoxic conditions, the HIF-1α protein uses molecular oxygen and is subjected to proteasomal degradation by proline hydroxylase domain protein. However, during hypoxia or ischemia, the degradation of HIF-1α is inhibited, causing its nuclear transfer and accumulation and inducing the transcriptional activation of specific target genes [8]. HIF-1α has been shown to regulate the proliferation and migration of CASMCs to mediate vascular remodeling [9] and plays a major role in vascular smooth muscle growth [10,11]. Hypoxia promotes HIF-1α expression in PASMCs of yak and llama, promoting PASMCs proliferation and migration to protect lungs from hypoxia-induced pulmonary hypertension [12,13]. Furthermore, hypoxia can activate the expression of HIF-1α, enhance the rate of neonatal rat aorta smooth muscle cell (NRSMC) proliferation, and reduce apoptosis [14]. Nonetheless, its potential role in the adaptation process of yak coronary artery to hypoxia needs to be further proved.

Autophagy is a highly conserved process, which mediates a series of enzymatic biological effects, such as cell proliferation, apoptosis, migration, vesicular transport, and other cellular processes. Autophagy induced by stress conditions such as hypoxia can provide energy for cells [15]. An increasing number of studies have shown that a high level of autophagy under hypoxia is a crucial process for cells to degrade and recycle their cellular components and organelles for self-preservation [16,17,18]. The activation of autophagy can prevent apoptosis induced by reactive oxygen species (ROS), reducing cardiac infarct size and improving left ventricular function to protect the heart from ischemia–reperfusion injury [19]. Autophagy is the key to inducing cardiac vascularization and inhibiting cardiomyocyte apoptosis after myocardial infarction [20]. Further, the autophagy signaling pathway PI3K/Akt regulates HIF-1α stability under hypoxic conditions, thereby controlling tumor-induced angiogenesis and metastasis [21]. These findings suggest that autophagy may play a crucial role in the adaptation of yaks to hypoxia.

Therefore, in this study, the expression and distribution of proliferation-associated genes, apoptosis, HIF-1α, and autophagy in heart tissues of yaks of different ages are analyzed. Meanwhile, the smooth muscle cells of the yak heart coronary artery were isolated and cultured in vitro to establish a hypoxia model to explore their adaptability to hypoxia and prove the potential role of HIF-1α and autophagy regulated by HIF-1α in this process. These results will contribute to understanding how the heart adapts to life in a hypoxic environment and may provide insights into the adaptation of the human heart at high altitudes.

## 2. Materials and Methods

### 2.1. Collection of Yak Heart Sample

The heart coronary artery samples of male yak from the same altitude slaughterhouse (Altitude > 4000 m) without clinical pathological manifestations were collected. The samples were collected at the following ages: 1–7 days ( n = 6), 6 months old (n = 6), 2 years old (n = 6), 4 years old (n = 6), 6 years old (n = 6), and 10 years old (n = 6). Some of the samples were stored at −80 °C for bioassays, whereas some were fixed using 4% Paraformaldehyde (PFA) for histological analyses. Some samples (6 months old) were placed for 4 h in sterile saline containing 100 IU/mL penicillin and 100 μg/mL streptomycin and transported back to the laboratory for tissue processing to isolate and culture yak heart CASMCs. All experimental procedures were handled according to the Animal Ethics Procedures and Guidelines of the People’s Republic of China. This study was also approved by the Animal Ethics Committee of Gansu Agricultural University (GSAU-Eth-VMC-2023-004).

### 2.2. Immunohistochemical (IHC) Analysis of Cardiac Tissue

Cardiac tissues were collected, fixed in 4% PFA solution, cut into 1 cm^3^ pieces, dehydrated, and embedded in paraffin wax. The paraffin-embedded tissues were sectioned into 4 μm thick slices, attached to coated sections according to conventional methods, deparaffinized from alcohol to water, and then heated in 10 mM citrate buffer for antigen repair (pH = 6.0). The cells were blocked with 3% H_2_O_2_ solution, incubated in a temperature chamber at 37 °C for 10 min, blocked using 5% goat serum working solution (SP kit A solution) at 37 °C for 15 min, and then incubated overnight with anti-HIF-1α (1:100, ab16066, Abcam, Cambridge, UK), PCNA (1:100, NBP1-32075, Novus Biologicals, Centennial, CO, USA), Bax (1:100, AF0120, Affinity, Liyang, China), and Bcl-2 (1:100, AF6138, Affinity) at 4 °C. For the negative control, primary antibody was replaced with phosphate-buffered saline (PBS, PH = 7.4). The sections were incubated with biotinylated goat anti-mouse IgG and goat anti-rabbit IgG (SP kit B solution) for 15 min, and then with streptavidin-conjugated horseradish peroxidase solution (SP kit C solution) for 10 min at 37 °C. Color was developed using 3, 3-diaminaniline (Bioss, Beijing, China) and counterstained with hematoxylin for 2 min. The hydrochloride alcohol was refined, followed by alcohol refinement and dehydration, and the resin seals were used. The images were captured on an Olympus-DP73 optical microscope (Olympus, Tokyo, Japan).

### 2.3. Culture of Primary Cells

CASMCs were cultured as described previously [22,23]. Briefly, the adventitia of blood vessels was removed, the isolated coronary arteries were opened, and endothelial cells were gently scraped off with a scalpel blade. The remaining tissues were cut into pieces of 1 mm^3^ size. The cells were incubated with 1 mg/mL collagenase type I (C0130, Sigma-Aldrich, St. Louis, MO, USA) and II (C6885, Sigma-Aldrich) in Dulbecco’s modified Eagles medium (DMEM/F12, 12400024, Gibco, Waltham, MA, USA). The cells were then collected through centrifugation at 1000 rpm for 5 min and resuspended with DMEM/F12 medium supplemented with penicillin (50 IU/mL), streptomycin (50 μg/mL), and fetal bovine serum (20% *v*/*v*, A5669701, Gibco). The cell suspension was filtered sequentially with 100, 200, and 400 μm cell strainers. Then, these cells were maintained at 37 °C in a 5% CO_2_ incubator. When primary cells grew to 90% confluency, they were seeded into a 6-well plate containing 2 mL of culture medium and allowed to grow. Prior to the treatment, cells were cultured in DMEM/F12 without serum for 12 h.

### 2.4. Cell Treatment

To investigate the effects of hypoxia on the expression of HIF-1α, PCNA, Bax, and Bcl-2 for different durations, we treated yak CASMCs with 5% O_2_ for different durations (0, 6, 12, 24, 36, 48, and 72 h). Cells were harvested to assess protein and mRNA levels.

The effects of hypoxia and normoxia on HIF-1α, PCNA, Bax, and Bcl-2 on the yak CASMCs treated with 5% O_2_ and 21% O_2_ for the same time (12, 24, 48, and 72 h) were compared. Cells were harvested to assess protein expression.

For inhibition studies, before hypoxia and normoxia treatment, the cells were treated with 200 μM HIF-1α activator dimethyloxallyl glycine (DMOG, HY-15893, MedChemExpress, Monmouth Junction, NJ, USA), 25 μM inhibitor (LW6, HY-13671, MedChemExpress), 20 μM Autophagy activator (RAPA, HY-10219, MedChemExpress), and 50 μM inhibitor (CQ, HY-17589A, MedChemExpress) for 2 h. Cells were harvested for assessing protein and mRNA expression.

### 2.5. Immunofluorescence (IF) Analysis

IF staining was used to evaluate and localize α-SMA, Calponin-1, and CD31 in yak CASMCs and the expression of LC3-II, P62 in the yak heart. The cells and heart tissue were fixed in 4% (*v*/*v*) paraformaldehyde for 1 h, incubated for 20 min with 0.5% Triton X-100 for permeabilization, and then blocked with 5% bovine serum albumin for 1 h. Then, primary anti-α-SMA (1:100, BF9212, Affinity), anti-Calponin-1 (1:100, bs-0095R, Bioss), and anti-CD31 (1:200, ab119339, Abcam, Cambridge, UK), anti-LC3-II (1:100, AF5402, Affinity), and P62 (1:100, 18420-1-AP, Proteintech, Wuhan, China) antibodies were added and incubated overnight at 4 °C. PBS was used as the negative control, and anti-mouse IgG Alexa Fluor 488 (1:1000, A32727, Thermo Fisher, Waltham, MA, USA) and anti-rabbit IgG Alexa Fluor 594 (1:1000, A32731, Thermo Fisher) were added for 1 h. Nuclei were counterstained with 4′,6-diamidino-2-phenylindole (DAPI). Digital images were acquired under a fluorescence inverted microscope (Olympus-DP71).

### 2.6. Cell Counting Kit-8 (CCK-8)

The cells of 3–4 passages in logarithmic growth phase were added to 96-well plates at a cell density of 3 × 10^4^, and four double wells were set in each group. The plates were placed into the incubator to culture for 24 h. The cells were treated with 5% O_2_ and 21% O_2_ for 12, 24, 48, and 72 h, and then 10 μL of CCK-8 solution and 90 μL of DMEM/F12 medium were added to the well plate and incubated for 2 h. The absorbance was determined at 450 nm using a microplate reader (SpectraMax i3x, Molecular Devices, Silicon Valley, NC, USA).

### 2.7. EdU Assay

The cells of 3–4 passages in logarithmic growth phase were inoculated in 6-well plates after adjusting the cell density to 1 × 10^5^ and cultured in the incubator until the normal growth stage. The cells were treated with hypoxia and normoxia, each for 12, 24, 48, and 72 h. An appropriate amount of 50 μM EdU was prepared by diluting the EdU with cell medium (1:1000), and then 50 μM EdU was added and incubated for 2 h. Cell fixative solution (PBS containing 4% PFA) was added for 30 min. The permeant (PBS (G0002-2L, Servicebio, Wuhan, China) containing 0.5% Triton X-100 (T8200, Solarbio, Beijing, China)) was added for 10 min. The click additive solution was added for 30 min in the dark. To visualize, 1 × Hoechst 33342 reaction solution was added to the cells and incubated for 30 min. Finally, the stained cells were observed and photos were taken for recording.

### 2.8. Flow Cytometry

The cells of 3–4 passages in the logarithmic growth phase were inoculated in 6-well plates and the cell density was adjusted to 1 × 10^5^. The cells were treated with hypoxia and normoxia for 12, 24, 48, and 72 h each. The cells were digested with trypsin and resuspended and counted in PBS. Cells were collected (2 × 10^5^), resuspended in 500 μL of 1 × Annexin V blinding buffer, and incubated with 5 μL of Annexin V-FITC and 5 μL of PI staining solution for 20 min. The proportion of apoptosis was immediately estimated by flow cytometry.

### 2.9. Tunel Assay

The cells of 3–4 passages in the logarithmic growth phase were inoculated in 6-well plates and the cell density was adjusted to 1 × 10^5^. The cells were treated with 5% O_2_, 21% O_2_, 5% O_2_ + LW6, 21% O_2_ + DMOG, 5% O_2_ + CQ, and 21% O_2_ + RAPA for 2 h each, operating according to the Tunel instructions. Then, 100 μL of prepared DNaseI reaction solution was added, and the solution was treated at 37 °C for 30 min. A total of 50 μL of TdT enzyme reaction solution was added drop by drop, and the reaction was carried out in a 37 °C wet box for 60 min. A total of 50 μL Streptavidin-TRITC labeling solution was added and the reaction was carried out at 37 °C in the dark for 30 min. A total of 1 µg/mL DAPI was added and stained for 10 min. Laser confocal fluorescence microscopy was used to observe and photograph. The excitation wavelength ranges from 450 nm to 500 nm, and the emission wavelength ranges from 515 nm to 565 nm (green fluorescence). A higher proportion of red fluorescence intensity to total cell fluorescence intensity indicates a higher level of apoptosis.

### 2.10. Detection of Autophagy

Ad-mRFP-GFP-LC3B was used to detect autophagy levels in CASMCs. CASMCs from yak heart were cultured until the degree of fusion reached 90%, washed with PBS, and then used for cell climbing with complete medium without double antibody. When the fusion degree of climb cells reached 40%, the cells were treated with 20 MOI Ad-mRFP-GFP-LC3B for 24 h. The treatment solution was discarded and fresh complete medium was added to continue the culture for another 24 h. Cells were treated with 5% O_2_, 21% O_2_, 5% O_2_ + LW6, 21% O_2_ + DMOG, 5% O_2_ + CQ, and 21% O_2_ + RAPA for 2 h. CASMCs were fixed with 4% paraformaldehyde for 6 h. Cells were stained with 1 µg/mL DAPI for 5 min. Observations were made using a laser confocal fluorescence microscope (DeltaVisionTMUltra, GE Healthcare Biosciences, Chicago, IL, USA) and photographed. After the occurrence of autophagy, Ad-mRFP-GFP-LC3B represented the autophagosome and gathered on the autophagosome membrane under the fluorescence microscope, showing yellow spots. More yellow fluorescent spots indicate more autophagosome numbers and higher autophagy levels. The average number of yellow fluorescent spots in the 10 fields of each treatment group was calculated to analyze the level of autophagy [24].

### 2.11. Cell Scratch Repair Assay

The cells of 3–4 passages in the logarithmic growth phase were inoculated into 6-well plates with the cell density adjusted to 1 × 10^5^. The cells were cultured until they were fused. A horizontal line was gently drawn in the well using a 200 μL gun head. The cells in the hypoxia group were placed in 5% O_2_, and they were placed in 21% O_2_ in the control group. The scratches of cells at different times were observed and photos were acquired for recording.

### 2.12. Western Blot

Cardiac tissue samples at different stages or harvested cells were lysed on ice using RIPA lysis buffer (Solarbio, Beijing, China) for 30 min, and protein concentrations were estimated by a BCA Protein Assay Kit (Thermo Fisher Scientific, MA, USA). Equivalent amounts of protein samples (40 μg) were loaded and separated using sodium dodecyl sulfate polyacrylamide gel electrophoresis, electrotransferred to a polyvinylidene difluoride membrane, and blocked with 5% skim milk for 2 h. Then, anti-HIF-1α (1:1000, ab16066, Abcam), anti-PCNA (1:1000, NBP1-32075, Novus Biologicals), anti-Bax (1:1000, AF0120, Affinity), anti-Bcl-2 (1:1000, AF6138, Affinity), anti-LC3-II (1:1000, AF5402, Affinity), anti-P62 (1:1000, 18420-1-AP, Proteintech), anti-Beclin1 (1:1000, AF5128, Affinity), and β-actin (1:8000, AF7018, Affinity) were added and incubated overnight at 4 °C. Next, secondary antibodies (horseradish peroxidase (HRP)-conjugated goat anti-mouse) (1:5000, HS201, TransGen, Beijing, China) or goat anti-rabbit (1:5000, HS101, TransGen) were added and incubated for 1 h. ECL Plus (Vazyme, Nanjing, China) was used to visualize the bands, and the experiment was repeated thrice. Image J 1.44P software was used to scan the Western blot bands for image analysis, and the relative expression was calculated. Original figures can be found in Supplementary Materials.

### 2.13. Quantitative Real-Time Polymerase Chain Reaction (qRT-PCR)

For qRT-PCR, cardiac tissue at different stages or harvested cells were lysed with TRIzol, and RNA concentration was measured. The RNA was reverse transcribed into cDNA and stored at −20 °C for later use. Primers for *HIF-1α*, *PCNA*, *Bax*, *Bcl-2*, *LC3-II*, *Beclin1*, *P62*, and *β-actin* (see Table 1 for Primer information) were designed employing Primer Premier 6.0 software (concentrations for all primers was 10 μM) and synthesized by Shanghai Sangon Gene Technology. qRT-PCR was performed using cDNA as template to amplify fragments of *HIF-1α*, *PCNA*, *Bax*, *Bcl-2*, *LC3-II*, *Beclin1*, *P62*, and *β-actin* using primers. The total reaction system of 20 μL contained cDNA (1 μL), primer (0.5 μL upstream and downstream), SYBR^®^Premix Ex Taq^TM^ II(AG11701, AG, Changsha, China) (10 μL), and sterile deionized water (8 μL). Reaction conditions were as follows: 40 cycles of pre-denaturation (95 °C) for 4 min, denaturation (95 °C) for 30 s, annealing (60 °C) for 30 s, and one cycle of extension (72 °C) for 20 s. qRT-PCR for each sample was repeated four times, and the relative expression was calculated using the 2^−^^ΔΔCt^ method based on the Cq value of each sample.

### 2.14. Statistical Analysis

All data are presented as the mean ± standard error of the mean of three independent experiments. All statistical analyses were performed using the GraphPad Prism 6.0 software (GraphPad software, San Diego, CA, USA). Significant differences were analyzed using unpaired Student’s *t*-test (two-tailed) or one-way analysis of variance. ^ns^ *p* ≥ 0.05 was not significant, 0.01 ≤ * *p* < 0.05 was significant, and ** *p* < 0.01 was extremely significant.

## 3. Results

### 3.1. Localization and Expression of Proliferation- and Apoptosis-Related Genes in Yak Heart at Different Developmental Stages

**Figure 1:**
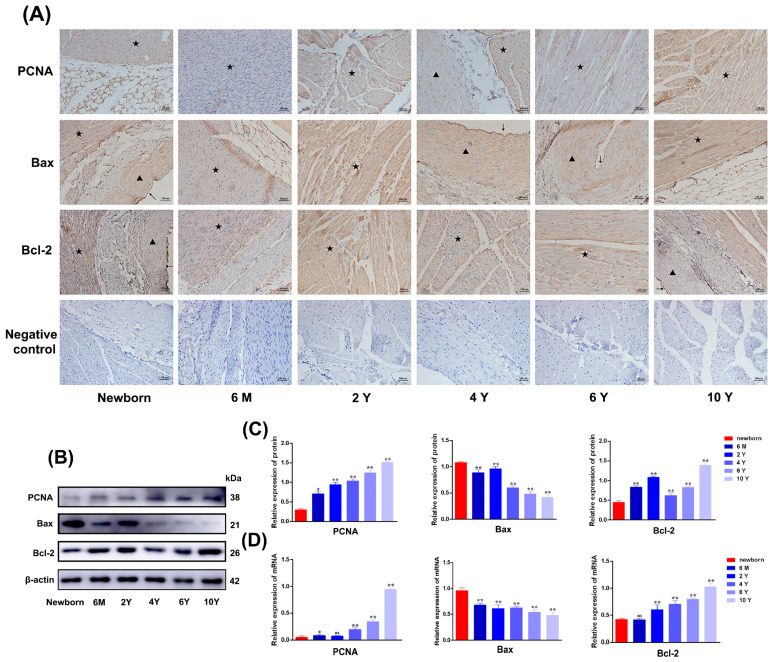
IHC, Western blot, and qRT-PCR analysis of changes in PCNA, Bax, and Bcl-2 in heart tissue of yak at different stages. (**A**) Localization and distribution of PCNA, Bax, and Bcl-2 proteins in yak hearts at different ages. Magnification: ×20, Bar = 100 μm. (**B**,**C**) The levels of PCNA, Bax, and Bcl-2 proteins in heart tissue of yaks at different ages were determined by Western blot. Western blot original images can be found in File S1. (**D**) The levels of *PCNA*, *Bax*, and *Bcl-2* mRNAs in heart tissues of yaks at different ages were determined by qRT-PCR. (^ns^ *p* ≥ 0.05, * *p* < 0.05, ** *p* < 0.01) (n = 3). ★: cardiomyocytes; ▲: vascular smooth muscle cells; ↑: vascular endothelial cells. Newborn: Newborn; 6 M: 6 months old; 2 Y: 2 years old; 4 Y: 4 years old; 6 Y: 6 years old; 10 Y: 10 years old.

To investigate whether proliferation- and apoptosis-related genes are involved in the adaptation of yak heart to hypoxic environments. Firstly, the localization of proliferation- and apoptosis-related genes in the hearts of yaks at different ages (Newborn, 6 months, 2 years, 4 years, 6 years, 10 years old) was detected. The results showed that PCNA was positively expressed at different ages in cardiomyocytes, vascular endothelial cells, and vascular smooth muscle cells of yak hearts. Bax and Bcl-2 were positively expressed at different ages in cardiomyocytes, vascular endothelial cells, and vascular smooth muscle cells of yaks (Figure 1A). Western blot analysis showed that the expression of PCNA protein in the heart increased significantly with the increase in the age of yak. The expression level of the pro-apoptotic protein (Bax) significantly decreased, while the expression level of the anti-apoptotic factor (Bcl-2) significantly increased (Figure 1B,C). The results of qRT-PCR analysis were basically consistent with the results of the Western blot (Figure 1D).

### 3.2. Hypoxia Affects the Expression of Genes Related to Proliferation and Apoptosis in Yak Heart CASMCs

**Figure 2:**
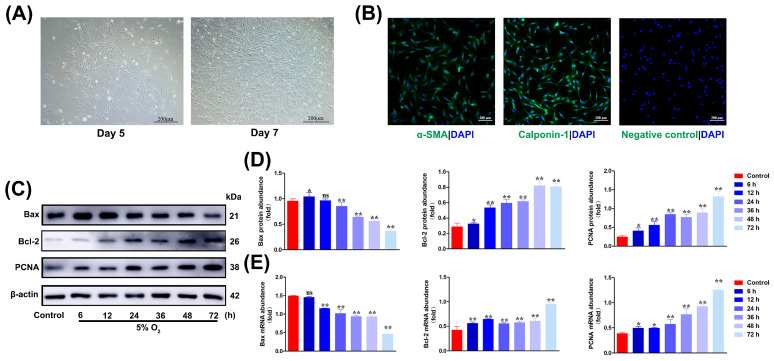
Hypoxia induces the expression of PCNA, Bax, and Bcl-2 in yak CASMCs. (**A**) Primary yak CASMCs were cultured in vitro. Magnification: ×10, Bar = 200 μm. (**B**) Identification of CASMCs in yak heart. Magnification: ×10, Bar = 200 μm. (**C**,**D**) Hypoxia-induced levels of PCNA, Bax, and Bcl-2 proteins in CASMCs of yak heart induced by Western blot. (**E**) Hypoxia-induced levels of *PCNA*, *Bax*, and *Bcl-2* mRNAs in CASMCs of yak heart by qRT-PCR. (^ns^ *p* ≥ 0.05, * *p* < 0.05, ** *p* < 0.01) (n = 3).

To investigate the effect of high-altitude hypoxia on proliferation- and apoptosis-related genes in cardiac vascular remodeling of yak, primary CASMCs were isolated and cultured in vitro. As shown in Figure 2A, the cultured cells began to adhere to the extension on the bottom of the culture flask within 5 days of the initial culture, and, after confluence (7–8 days of culturing), they exhibited a typical “hills and valleys” appearance. The purification of primary cells was confirmed through strongly positive staining for α-SMA (green) and Calponin-1 (green) but negative staining for CD31. Therefore, IF staining confirmed that more than 98% of the isolated cells were indeed smooth muscle cells (Figure 2B).

The hypoxia-induced proliferation of CASMCs is a key step in hypoxic cardiac vascular remodeling. To test the effect of moderate hypoxia on the mitogenic proliferation and apoptosis of CASMCs, primary cultured CASMCs were exposed to hypoxia (5% O_2_) for 0, 6, 12, 24, 36, 48, and 72 h, respectively. The expression of PCNA, Bax, and Bcl-2 proteins was detected. As shown in Figure 2C,D, PCNA protein expression was induced after 24 h of hypoxia, and the proliferation of CASMCs increased with time. Subsequently, the expression of Bax and Bcl-2 proteins to hypoxia for 0, 6, 12, 24, 36, 48, and 72 h were evaluated, and it was found that the expression of Bax was down-regulated and the expression of Bcl-2 was up-regulated under hypoxic conditions. The results of qRT-PCR analysis were consistent with the results of Western blot analysis (Figure 2E).

### 3.3. The Expression of PCNA, Bax, and Bcl-2 Proteins in Coronary Smooth Muscle Cells of Yak Heart Under Different O_2_ Concentrations at the Same Time

**Figure 3:**
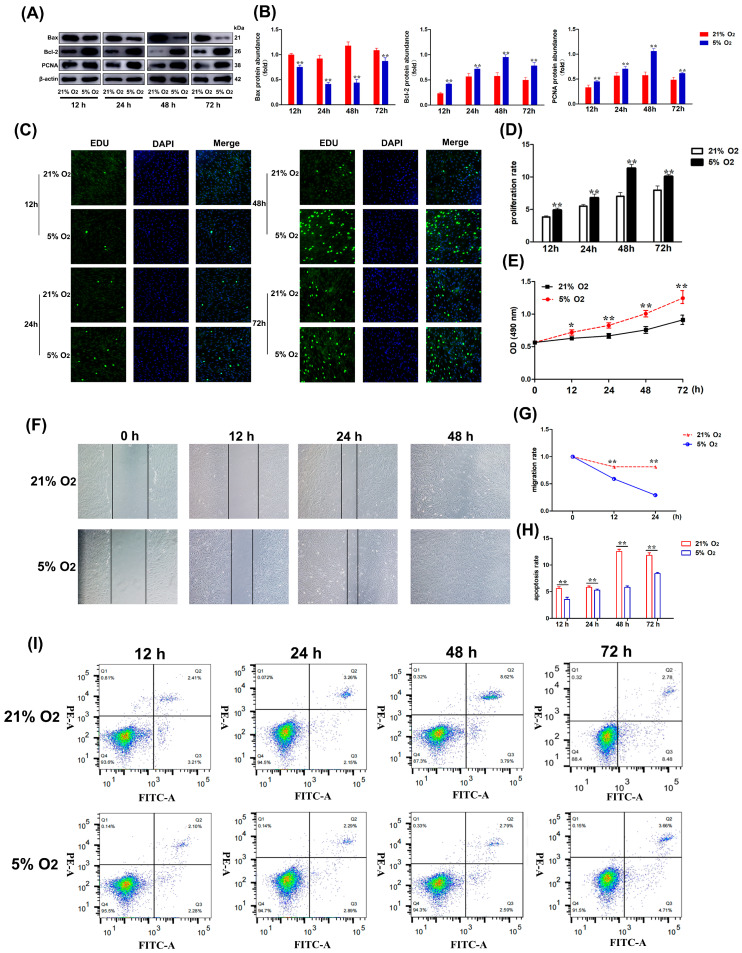
Expression levels of proliferation and apoptosis in CASMCs of yak heart at different O_2_ concentrations at the same time. (**A**,**B**) The levels of PCNA, Bax, and Bcl-2 proteins in CASMCs at 5% O_2_ and 21% O_2_ at the same time. (**C**,**D**) EdU proliferation assay was used to detect the fluorescence intensity of yak heart CASMC proliferation under 21% O_2_ or 5% O_2_ conditions. Magnification: ×10, Bar = 200 μm. (**E**) CCK-8 proliferation assay was used to detect the proliferation of CASMCs in yak heart under 21% O_2_ (black) or 5% O_2_ (red) conditions. (**F**,**G**) Effect of hypoxia (5% O_2_) on the migration of yak heart CASMCs (n = 3). Magnification: ×10, Bar = 200 μm. (**H**,**I**) The proliferation of CASMCs in yak heart was detected by Annexin V-FITC/PI apoptosis assay under 21% O_2_ or 5% O_2_ conditions. ( * *p* < 0.05, ** *p* < 0.01) (n = 3).

The expression of PCNA, Bax, and Bcl-2 in yak CASMCs under 5% O_2_ and 21% O_2_ conditions was examined. Western blot results showed that compared with the 21% O_2_ condition, 5% O_2_ promoted the expression of PCNA protein, and the level of Bax protein was significantly decreased, while that of Bcl-2 was significantly increased. The difference was the most significant at 48 h (Figure 3A,B). The EdU proliferation test measures cell proliferation. The nuclei in blue cells were stained by Hoechst 33342 and the proliferative cells stained green by EdU, while the cells that appeared pink after merging the images were the proliferating cells. With the 21% O_2_ treatment group, the cells in the 5% O_2_ treatment group showed significantly increased proliferation (Figure 3C,D). Likewise, the results of the CCK-8 cell proliferation kit used to measure cell proliferation showed that compared with the 21% O_2_ treatment group, the rate of the 5% O_2_ treatment group was significantly higher, the cell proliferation significantly increased after 24 h of treatment, and the difference was significant after 48 h, which was consistent with the expression of the EdU and PCNA protein (Figure 3E). In addition, the yak heart CASMCs in 5% O_2_ and 21% O_2_ treatment groups were scratched. The wound healing scratch experiment results show that the migration distance of cells is reduced after 12 h of scratch treatment. Compared with the control group in the 21% O_2_ concentration environment, the cell migration speed of the treatment group in the 5% O_2_ concentration environment was significantly faster. (Figure 3F,G). The Annexin V-FITC/PI apoptosis assay used to detect apoptosis revealed that compared with the 21% O_2_ treatment group, the apoptosis rate of the 5% O_2_ treatment group was significantly decreased after 24 h, and the difference was significant at 72 h (Figure 3H,I).

### 3.4. Hypoxia Induces the Expression of HIF-1α Gene in CASMCs of Yak Heart

**Figure 4:**
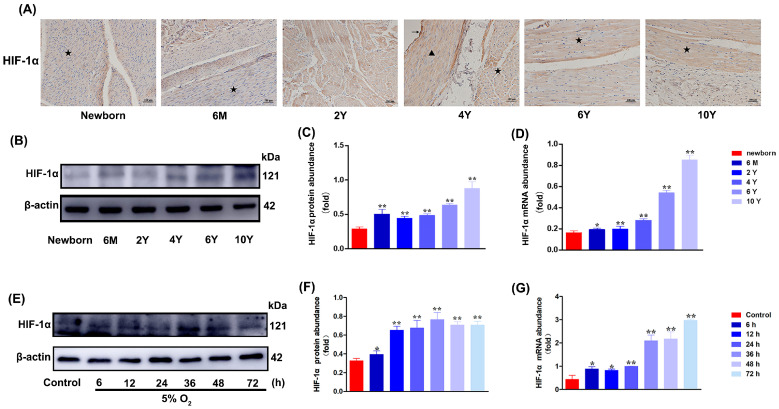
Analysis of HIF-1α expression in yak heart tissue at different developmental stages by IHC, Western blot, and qRT-PCR. (**A**) Localization and distribution of HIF-1α protein in yak hearts at different ages. Magnification: ×20, Bar = 100 μm. (**B**,**C**) The levels of HIF-1α protein in heart tissue of yaks at different ages were determined by Western blot. 6 M: 6 months old; 2 Y: 2 years old; 4 Y: 4 years old; 6 Y: 6 years old; 10 Y: 10 years old. (**D**) The levels of *HIF-1α* mRNA in heart tissue of yaks at different ages were determined by qRT-PCR. (**E,F**) Hypoxia-induced HIF-1α protein level in yak heart CASMCs. (**G**) Hypoxia-induced *HIF-1α* mRNA level in CASMCs of yak heart. The same protein (HIF-1α) at different ages. ( * *p* < 0.05, ** *p* < 0.01) (n = 3). ★: cardiomyocytes; ▲: vascular smooth muscle cells; ↑: vascular endothelial cells. Newborn: Newborn; 6 M: 6 months old; 2 Y: 2 years old; 4 Y: 4 years old; 6 Y: 6 years old; 10 Y: 10 years old.

To investigate whether the HIF-1α gene is involved in the adaptation of yak hearts to hypoxic environments, first, the localization of the HIF-1α gene in yak hearts at different ages was determined. HIF-1α was positively expressed at different ages in cardiomyocytes, vascular endothelial cells, and vascular smooth muscle cells of yak hearts (Figure 4A). Its protein and mRNA expression levels increased with age (Figure 4B–D). The primary cultured CASMCs were exposed to hypoxia (5% O_2_) for 0, 6, 12, 24, 36, 48, and 72 h, respectively, and the expression of HIF-1α protein was detected. As shown in Figure 4E–G, the Western blot and qRT-PCR results showed that the expression levels of HIF-1α protein and mRNA in yak heart CASMCs increased with time, and the trend was basically consistent.

### 3.5. DMOG Activation and LW6 Inhibition of HIF-1α Affect the Proliferation and Anti-Apoptotic Ability of CASMCs

**Figure 5:**
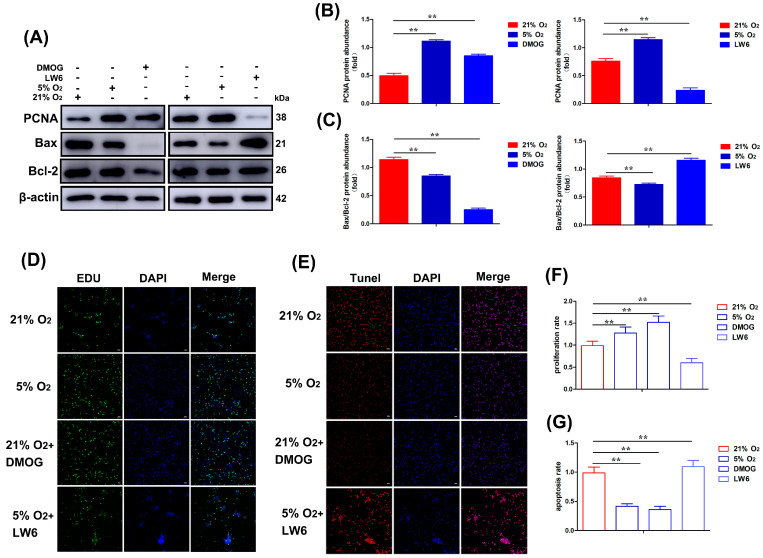
Activation (DMOG) (200 μM) and inhibition (LW6) (25 μM) of HIF-1α affected the proliferation and anti-apoptotic ability of yak heart CASMCs. (**A**–**C**) Effects of DMOG and LW6 on the expression of proliferation- and apoptosis-related proteins in yak heart CASMCs. (**D**,**F**) EdU proliferation was used to detect the effect of DMOG and LW6 on the proliferation expression of yak heart CASMCs. (**E**,**G**) Tunel apoptosis assay was used to detect the effect of DMOG and LW6 on apoptosis expression in CASMCs of yak heart. Magnification: ×10, Bar = 200 μm. ( ** *p* < 0.01) (n = 3).

HIF-1α is a master regulator of transcription in hypoxia cells. To further investigate the effects of activating or inhibiting the hypoxia pathway on the expression of factors related to the proliferation and apoptosis of yak cardiac CASMCs, we treated CASMCs with dimethoxyaldehyde glycine (DMOG, a competitive inhibitor of prolyl hydroxylase as well as inducer of HIF-1α) (200 μM) for 2 h at 21% O_2_. Western blot results showed that DMOG treatment increased the protein level of PCNA, the expression level of the Bax/Bcl-2 ratio was down-regulated, LW6 (25 μM) could reduce PCNA expression without affecting HIF-1α expression, and the expression of Bax/Bcl-2 ratio was upregulated (Figure 5A–C). The cells treated with 21% O_2_, 5% O_2_, 21% O_2_ + DMOG, and 5% O_2_ + LW6 were measured by the EdU proliferation test. The results showed that the cell proliferation of the 5% O_2_ and 21% O_2_ + DMOG treatment groups was significantly increased compared with the 21% O_2_ treatment group. The cells in the 5% O_2_ + LW6 treatment group were significantly reduced (Figure 5D,F). The Tunel assay showed that compared with the 21% O_2_ treatment group, the apoptosis rate of 5% O_2_ and 21% O_2_ + DMOG treatment group was significantly decreased, and the apoptosis rate of 5% O_2_ + LW6 treatment group was significantly increased (Figure 5E,G).

### 3.6. DMOG Activation and LW6 Inhibition of HIF-1α Affect the Autophagy Ability of Yak Heart CASMCs

**Figure 6:**
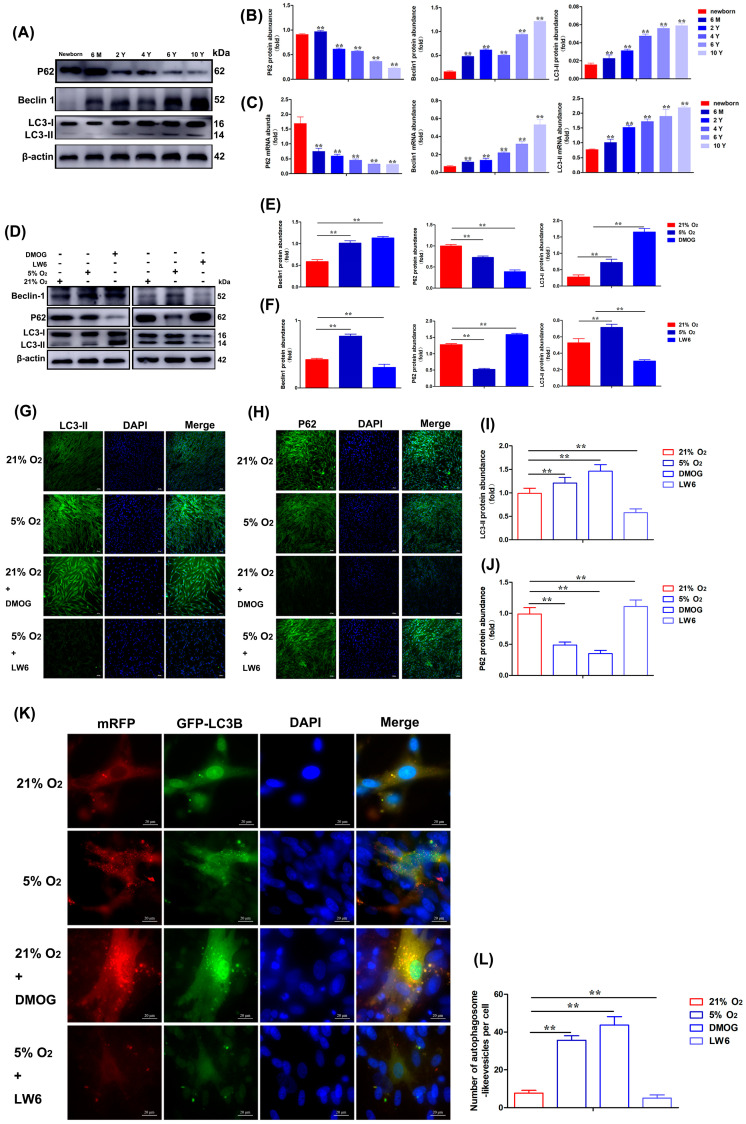
Activation of DMOG (200 μM) and inhibition of HIF-1α by LW6 (25 μM) affected the autophagic ability of yak heart CASMCs. (**A**,**B**) The levels of P62, Beclin1, and LC3-II proteins in heart tissue of yaks at different ages were determined by Western blot. (**C**) The levels of *P62*, *Beclin1*, and *LC3-II* mRNAs in heart tissue of yaks at different ages were determined by qRT-PCR. (** *p* < 0.01) (n = 3). 6 M: 6 months old; 2 Y: 2 years old; 4 Y: 4 years old; 6 Y: 6 years old; 10 Y: 10 years old. (**D**–**F**) Effects of DMOG and LW6 on the expression of autophagy-related proteins in yak heart CASMCs. (**G**–**J**) The effects of DMOG and LW6 on the expression of LC3-II and P62 proteins in yak heart CASMCs by IF analysis. Magnification: ×10, Bar = 200 μm. (**K**,**L**) The effect of DMOG and LW6 on autophagy expression in yak heart CASMCs by Ad-mRFP-GFP-LC3B analysis. Bar = 20 μm. (** *p* < 0.01) (n = 3).

To investigate whether hypoxia is involved in the adaptation of yak hearts to hypoxic environments through the autophagy pathway, Western blot analysis showed that the expression of LC3-II and Beclin1 increased significantly in the coronary artery with an increase in the age of yak. However, the expression of P62 decreased with age (Figure 6A,B). The results of qRT-PCR analysis were basically consistent with that of the Western blot (Figure 6C). To investigate whether hypoxia affected the expression of autophagy-related genes in yak heart CASMCs, the Western blot results showed that DMOG (200 μM) increased the level of LC3-II protein. Then, we verified that DMOG had a similar regulatory effect on Beclin1 expression in CASMCs, and P62 expression was down-regulated (Figure 6D,E). LW6 (25 μM) decreased the expression levels of LC3-II protein and Beclin1 protein. The expression of P62 was increased (Figure 6D,F). The IF results showed that the fluorescence intensity of LC3-II expression in the LW6 treatment group was significantly weakened compared with the 21% O_2_ treatment group, and the fluorescence intensity of LC3-II expression in the 5% O_2_ and DMOG addition was significantly increased (Figure 6G,I). Meanwhile, compared with the 21% O_2_ treatment group, the fluorescence intensity of P62 expression in the LW6 treatment group was significantly enhanced, and the fluorescence intensity of the P62 expression in 5% O_2_ and DMOG was significantly weakened (Figure 6H,J). As an autophagy marker, Ad-mRFP-GFP-LC3B is widely used to assess autophagy levels. The cells were introduced 2 h before cell treatment and subsequently treated with 21% O_2_, 5% O_2_, 21% O_2_ + DMOG, and 5% O_2_ + LW6. The results showed that the number of yellow fluorescent spots of Ad-mRFP-GFP-LC3B decreased in the LW6 treatment group compared with the 21% O_2_ treatment group, and the addition of 5% O_2_ and 21% O_2_ + DMOG increased the number of yellow fluorescent spots (Figure 6K,L).

### 3.7. RAPA Activation and CQ Inhibition of Autophagy Affect the Autophagy, Proliferation, and Anti-Apoptosis Ability of CASMCs

**Figure 7:**
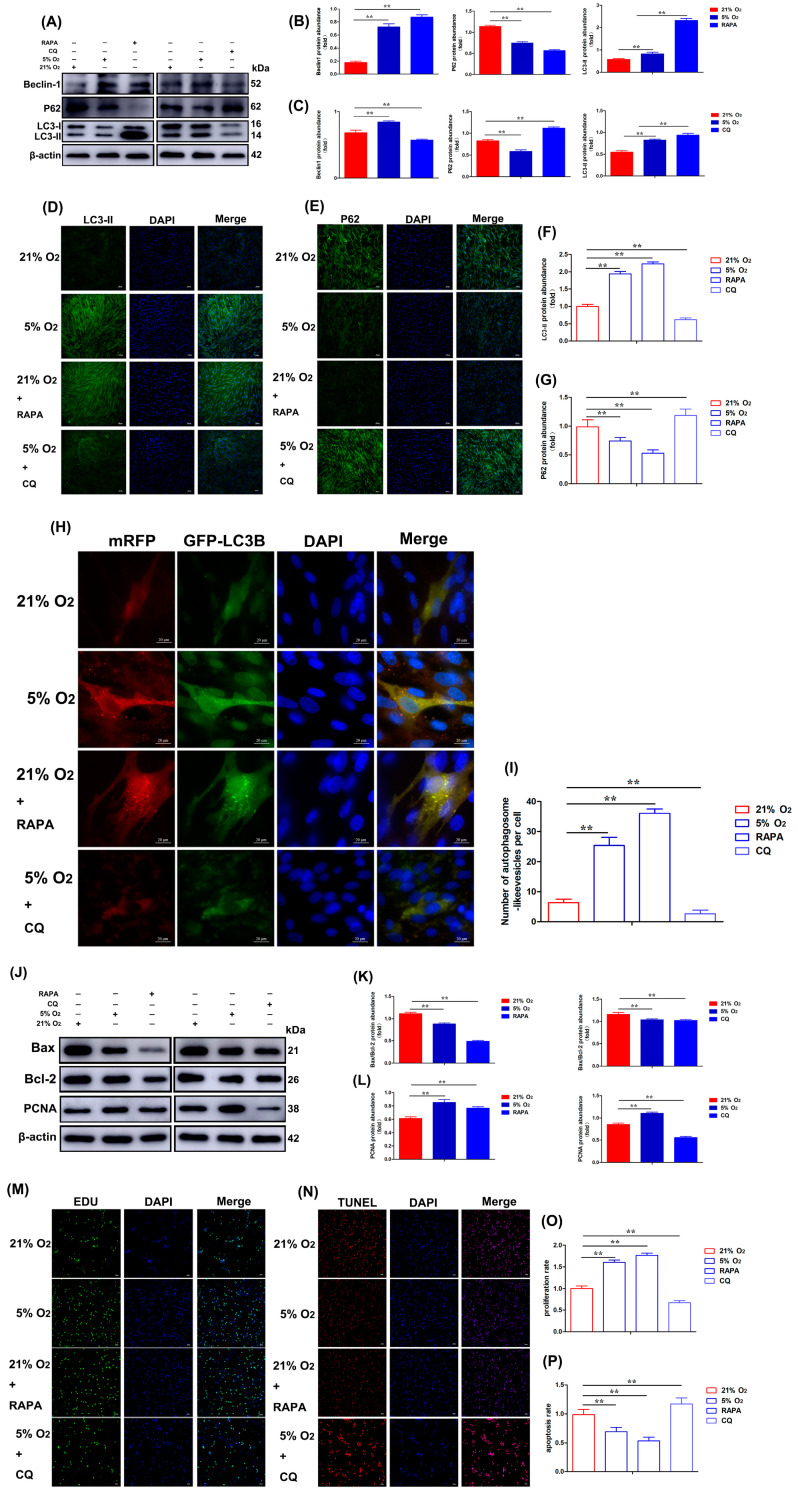
Activation (RAPA) (20 μM) and inhibition (CQ) (50 μM) of HIF-1α affected autophagy, proliferation, and anti-apoptosis ability of yak heart CASMCs. (**A**–**C**) Effects of RAPA and CQ on the expression of autophagy-related proteins in yak heart CASMCs. (**D**–**G**) RAPA and CQ on the expression of LC3-II and P62 protein in yak heart CASMCs by IF analysis. Magnification: ×10, Bar = 200 μm. (**H**,**I**) The effects of RAPA and CQ on autophagy expression in yak heart CASMCs by Ad-mRFP-GFP-LC3B analysis. Bar = 20 μm. (**J**–**L**) The effects of RAPA and CQ on the expression of proliferation- and apoptosis-related proteins in yak heart CASMCs by Western blot. (**M**,**O**) EdU proliferation was used to detect the effect of RAPA and CQ on proliferation expression in yak heart CASMCs. (**N**,**P**) Tunel apoptosis was used to detect the effect of RAPA and CQ on apoptosis expression in yak heart CASMCs. Magnification: ×10, Bar = 200 μm. (** *p* < 0.01) (n = 3).

Autophagy is an evolutionarily conserved process for the turnover of intracellular materials in eukaryotes. To further investigate the effect of the autophagy pathway on the expression of autophagy-, proliferation-, and anti-apoptosis-related genes in yak heart CASMCs, we treated yak heart CASMCs with rapamycin (RAPA, an autophagy activator) (20 μM) for 2 h at 21% O_2_. The Western blot results showed that RAPA increased LC3-II and Beclin1 protein levels, while the expression of P62 was down-regulated (Figure 7A,B). However, CQ (50 μM) decreased the expression levels of LC3-II and Beclin1 proteins, and increased the expression of P62 (Figure 7A,C). The IF results showed that the fluorescence intensity of LC3-II expression in the CQ treatment group was significantly weakened compared with the 21% O_2_ treatment group, and the fluorescence intensity of LC3-II expression in the 5% O_2_ and RAPA treatment group was significantly increased (Figure 7D,F). However, the fluorescence intensity of P62 expression was exactly the opposite (Figure 7E,G). The Ad-mRFP-GFP-LC3B results showed that the number of yellow fluorescent spots of Ad-mRFP-GFP-LC3B decreased in the CQ treatment group compared with the 21% O_2_ treatment group, and the treatment group of 5% O_2_ and RAPA both increased the number of yellow fluorescent spots (Figure 7H,I).

Meanwhile, to further detect the effect of autophagy on the expression of genes related to proliferation and apoptosis in yak heart CASMCs. The results showed that RAPA could increase PCNA protein levels. The ratio of Bax/Bcl-2 expression was down-regulated (Figure 7J,K). CASMCs were treated with CQ for 48 h under hypoxic conditions. The expression of PCNA was down-regulated and the ratio of Bax/Bcl-2 expression was up-regulated (Figure 7J,L). The cells treated with 21% O_2_, 5% O_2_, 21% O_2_ + RAPA, and 5% O_2_ + CQ were measured by the EdU proliferation test. The results showed that compared with the 21% O_2_ treatment group, the cell proliferation of the 5% O_2_ and RAPA treatment group was significantly increased, and the cell proliferation of the CQ treatment group was significantly decreased (Figure 7M,O). The Tunel assay showed that compared with the 21% O_2_ treatment group, the apoptosis rate of the RAPA and 5% O_2_ treatment group were significantly decreased, and the apoptosis rate of the CQ treatment group was significantly increased. The results suggested that 5% O_2_ and RAPA had a certain inhibitory effect on the apoptosis of CASMCs (Figure 7N,P).

## 4. Discussion

Hypoxia-regulated proliferation, migration, and apoptosis of VSMCs are crucial in inducing cardiovascular remodeling [25]. The proliferation and migration of yak heart vessels is a non-pathological adaptive change in yak heart vessels to adapt to the hypoxic environment and avoid pulmonary hypertension and right heart failure due to hypoxia [26]. Previous studies on age-related changes in the structure and associated factors of yak heart [27] and lung [28] have revealed that their special structures have a key role in adaptation to hypoxia. Previous studies have shown that low-altitude animals can also cause changes in cell proliferation and autophagy in low-oxygen environments, but this will cause more pathological changes in the body, even irreversible damage [29]. For yaks living in high-altitude areas for a long time, their heart and lungs show changes in cell proliferation, anti-apoptosis, and other results after long-term adaptation. However, this is more of an adaptive change and does not cause pathological damage [30]. In this study, the proliferation rate of the yak heart coronary artery increased with age. In addition, the in vitro study showed that with the increase in treatment time, there was a significant increase in the proliferation of in vitro-cultured CASMCs in yak hearts, and the apoptosis rate decreased significantly. It has been suggested that survivin can inhibit apoptosis by interacting with the gene of apoptosis. The high expression of survivin in the adult yak heart can inhibit cardiomyocyte apoptosis [31] and help to adapt to the hypoxic environment. In addition, it has been confirmed that hypoxia has pro-migration and anti-apoptosis effects on human umbilical vein endothelial cells [32]. Prolonged hypoxia also promoted changes in renal interstitial fibroblasts’ cellular phenotype, promoting the proliferation and activation in yak [33]. In the present study, Hypoxia caused an anti-apoptosis effect in the coronary artery of the yak heart, which may be an important mechanism of physiological hypoxic adaptation in the yak heart.

To further evaluate hypoxia as a potential key factor in regulating proliferation and anti-apoptosis in VSMCs of yak hearts, yak heart CASMCs were cultured in vitro in this study and subjected to hypoxic conditions for different durations. The results showed an increase in the rate of cell proliferation. Studies have demonstrated that CASMCs undergo changes such as cell hypertrophy, proliferation, phenotypic transformation, and migration during vascular remodeling, inducing intimal hyperplasia, and consequently leading to arterial stenosis [34]. Likewise, hypoxic stimulation can also significantly promote the proliferation of NRSMCs in a time-dependent manner, with a decline in the rate of apoptosis [14]. Isolated PASMCs exhibit an enhanced rate of proliferation and resistance to apoptosis under hypoxic conditions, possibly contributing to vascular remodeling [35,36]. Thus, in this study, hypoxia promoted the proliferation of CASMCs, which may be an important reason for the proliferation of yak coronary arteries with the increase in age. These results suggest that the proliferation of cardiac coronary arteries is a key factor for the yak heart to undergo hypoxic adaptation. In addition, while proliferation occurred, apoptosis was inhibited under hypoxic conditions. In addition, while proliferation occurred, apoptosis was inhibited under hypoxic conditions. Vascular cell proliferation and apoptosis resistance are important changes in vascular remodeling [37]. Thus, we hypothesize that the proliferation of CASMCs increased and the distance of migration was significantly reduced under hypoxic conditions.

HIF-1α can aid in adjusting to reduced oxygen availability by regulating the expression of corresponding target genes and is also crucial in regulating the proliferation, migration, and anti-apoptosis of CASMCs in the early stage of vascular development [38,39]. This study demonstrates that HIF-1α expression increased significantly with the increase in yak age and is regulated by hypoxia. As an inducer of HIF-1α, DMOG is a competitive inhibitor of the asparagine inhibitor hydroxylase, effectively inhibiting the inactivation of endogenous HIF and up-regulating the expression of HIF-1α [40]. DMOG sometimes shows opposite effects such as the inhibition of proliferation. The reasons for this result are various. The first is the difference in cell type, and tumor cells and normal cells will have different effects on DMOG treatment [41]. In addition, the concentration and action time of DMOG will also affect its effect. A low concentration of DMOG will promote cell adaptation to hypoxia, a high concentration of DMOG will lead to cell signal imbalance, a short time of action will start the normal cell adaptation mechanism, and the continuous activation of HIF-1α will consume excessive cell energy. There are also oxygen concentrations and other signaling molecules that can make a difference [42,43]. Similar to hypoxic conditions, DMOG increased the HIF-1α expression, promoted the proliferation of CASMCs, and reduced cell apoptosis. On the contrary, LW6 inhibited HIF-1α expression in hypoxia-induced A549 cells, inducing hypoxia-selective apoptosis and a decrease in mitochondrial membrane potential [44]. The results of our current study are consistent with those published earlier, showing that LW6 inhibited HIF-1α expression and cell proliferation in CASMCs and promoted cell apoptosis. Therefore, our findings on the effects of HIF-1α on the proliferation, apoptosis, and migration of yak CASMCs are consistent with related reports on mice, rats, and humans [45,46,47,48]. However, some studies have reported that the DMOG-induced enhanced expression of HIF-1α inhibits the hypoxia-induced proliferation of vascular smooth muscle cells and right ventricular remodeling [49,50]. The results suggested that hypoxia can mediate HIF-1α expression and play a role in the proliferation, migration, and apoptosis of CASMCs. This revealed that HIF-1α plays an important role in the hypoxic adaptation process of the yak heart coronary artery.

The hypoxia-mediated regulation of autophagy level is essential for cell survival [51]. Autophagy, which is the degradation and recycling process of intracellular macromolecules and damaged organelles, is highly conserved across all eukaryotes and is essential for the maintenance of normal cardiac function [52,53]. Autophagy involves multiple signaling pathways and regulatory genes in the endothelial cells of pulmonary arteries and PASMCs and is important in the occurrence and development of pulmonary arterial hypertension (PAH) [54]. When exploring the hypoxia-induced autophagy in yak hearts, conventional cells usually start the autophagy mechanism when the oxygen content is significantly lower than the normal level, and the degree of autophagy will gradually increase with the deepening of hypoxia. However, yaks have evolved physiological characteristics that are highly adapted to low oxygen since they have lived in high-altitude and low-oxygen environments for a long time [55]. In this study, it was found that yak heart CASMCs may initiate the autophagy response at a relatively low oxygen concentration, and the initiation rate of autophagy is faster. At the same time, the activation degree of autophagy in yak heart cells may be significantly higher than that in conventional cells under the same hypoxic conditions, which indicates that yak heart cells have a more sensitive and efficient hypoxic autophagy response mechanism to ensure normal physiological function and metabolic demand of the heart under long-term hypoxic environments. The occurrence of autophagy in conventional cells under hypoxic conditions shows a certain time regularity. Usually, the level of autophagy gradually increases in the early stage of hypoxia and then gradually decreases with the extension of time after reaching the peak [56]. However, the temporal dynamic changes in autophagy in yak heart cells under a hypoxic environment may be different from that in conventional cells. Our study found that the autophagy level of yak heart CASMCs can be maintained at a relatively stable high state under long-term hypoxic stimulation, indicating that yak heart cells have stronger self-regulation ability and can continue to cope with long-term hypoxic challenge through autophagy to ensure the normal function of the heart. Accordingly, this study demonstrates that with age and under the condition of hypoxia, autophagy was induced and its levels increased in the coronary artery of the yak heart. The autophagy activator RAPA promoted hypoxia cardiac vascular remodeling and cell proliferation and enhanced anti-apoptosis and other cellular processes. The autophagy inhibitor CQ inhibits cell proliferation and anti-apoptotic processes. Studies have demonstrated that in animals exposed to ischemia–reperfusion injury (I/R), higenamine can reduce the size of myocardial infarction and apoptosis; this effect can be mitigated by PI3K/Akt pathway inhibitors. Higenamine may improve cardiac function after I/R injury by regulating the PI3K/Akt pathway [57]. Further, the TP53-induced regulator of glycolysis and apoptosis (TIGAR) inhibits the proliferation and migration of PASMCs by inhibiting autophagy, thereby improving hypoxia-induced PAH [58]. Thus, autophagy potentially plays a key role in promoting cell survival and anti-apoptosis under hypoxic conditions. In addition, cells fail to maintain adequate antioxidant capacity under hypoxic conditions, resulting in ROS accumulation. Autophagy can prevent the accumulation of ROS by eliminating damaged mitochondria [59]. Combined with the results of this study, animals living at low altitudes can also cause changes in cell proliferation and autophagy in low-oxygen environments, but this will cause more pathological changes in the body, even irreversible damage. For yaks living in high-altitude areas for a long time, their heart and lungs show changes in cell proliferation, anti-apoptosis, and other results after long-term adaptation. However, this is more of an adaptive change and does not cause pathological damage. Autophagy, apoptosis, and proliferation interact to participate in functional regulation during the hypoxic adaptation of the yak heart, which is the key factor for yaks to adapt to high-altitude environments.

## 5. Conclusions

In conclusion, our study demonstrated that hypoxia promotes the proliferation, migration, and apoptotic resistance of CASMCs. Concurrently, the addition of the HIF-1α agonist, DMOG, promoted the proliferation of CASMCs and inhibited CASMC apoptosis, while the HIF-1α inhibitor (LW6) down-regulated the proliferation and promoted the apoptosis of CASMCs. Furthermore, the activation (with RAPA treatment) or inhibition (with CQ treatment) of autophagy was consistent with the regulation of expression by hypoxia. The results revealed that the increase in the yak heart coronary artery with age increases cell proliferation and migration, mainly achieved through hypoxia-mediated HIF-1α-regulated autophagy. These results would contribute to understanding how the heart adapts to life in a hypoxic environment. This study only illustrates the process of cardiac hypoxia adaptation through the cellular level but it has certain limitations at the animal level, which is also the direction of our future research and development. Given the significance of hypoxia in pulmonary hypertension and atherosclerosis, our findings may provide some essential information for the treatment of conditions associated with plateau medicine.

## Figures and Tables

**Table 1:** Primers used in qRT-PCR.

Genes	Primer Sequences (5′–3′)	Length (bp)	Accession No.
*HIF1A*	F:CTACATTACCTGCCTCTGAAACTCC	178	XM_005890694.1
	R:ACGCTTTGTCTGGTGCTTCC		
*PCNA*	F:CTCGTCTCATGTCTCCTTGGT	137	XM_005906528.2
	R:TGTCTTCATTGCCAGCACATT		
*BAX*	F:TTTGCTTCAGGGTTTCATC	174	XM_014478123.1
	R:CAGCTGCGATCATCCTCT		
*BCL2*	F:CCTGTGGATGACCGAGTACC	143	XM_005910188.1
	R:TGAGCAGTGCCTTCAGAGAC		
*MAP1LC3B*	F:TGTTAGGTCAGGCAGTCA	150	NM_001001169.1
	R:GTAGTAGGAAGCACTCGTTA		
*BECN1*	F:AACCTCAGCCGAAGACTA	253	NM_001033627.2
	R:TCAGCCTCTCCTCCTCTA		
*P62*	F:ATCAGCCTCTGGTCCATC	128	NM_001205519.1
	R:TTCTCTTGCCTCCGTGTT		
*β-actin*	F:TCCTGCGGCATTCACGAAACTAC	80	XM_005887322.2
	R:GTGTTGGCGTAGAGGTCCTTGC		

## Data Availability

The data that support the study findings are available upon request.

## Supplementary Materials

The following supporting information can be downloaded at: https://www.mdpi.com/article/10.3390/biom15020256/s1, File S1: Western blot original images.

## Abbreviations

The following abbreviations are used in this manuscript:

HIF-1α	Hypoxia-inducible factor-1α
CASMCs	Coronary vascular smooth muscle cells
ROS	Reactive oxygen species
PFA	Paraformaldehyde
IHC	Immunohistochemical
IF	Immunofluorescence
DMOG	Dimethyloxallyl glycine
RAPA	Rapamycin
CQ	Chloroquine
CCK-8	Cell Counting Kit-8
EdU	5-ethynyl-2-deoxyuridine
V-FITC/PI	Annexin V-fluorescein isothiocyanate/propidium iodide
DAPI	4′,6-diamidino-2-phenylindole
DAB	3, 3-diaminaniline
PBS	Phosphate-buffered saline
