# Supplementary material for: Hypoxia Regulates the Proliferation and Apoptosis of Coronary Artery Smooth Muscle Cells Through HIF-1α Mediated Autophagy in Yak
Source: Biomolecules. 2025 Feb 10;15(2):256. doi: 10.3390/biom15020256 (PMC11853270; doi:10.3390/biom15020256)

Figure 1B

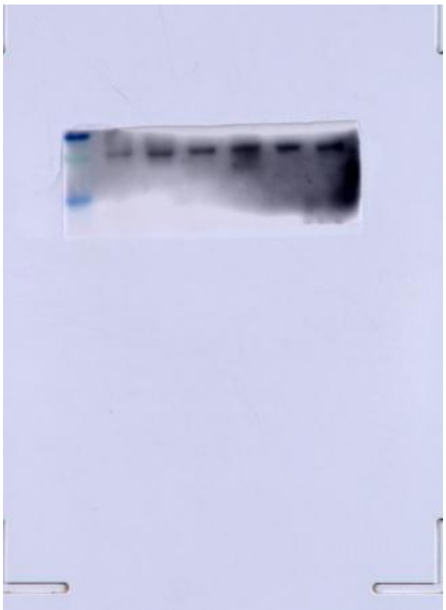

PCNA

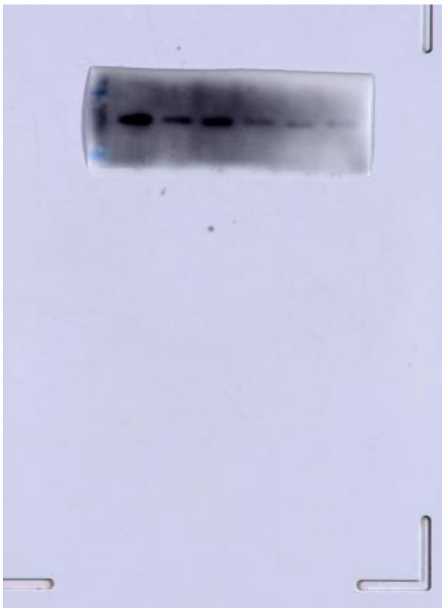

Bax

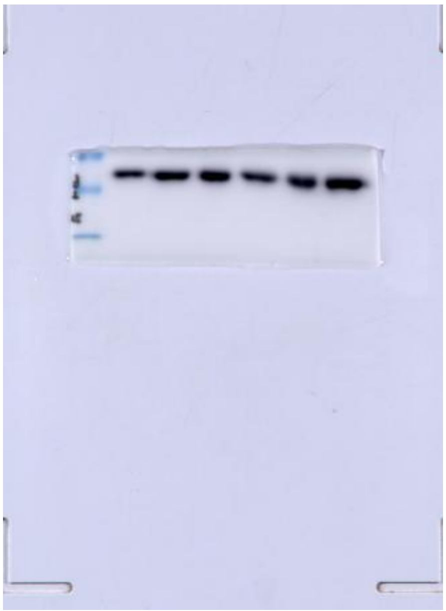

Bcl-2

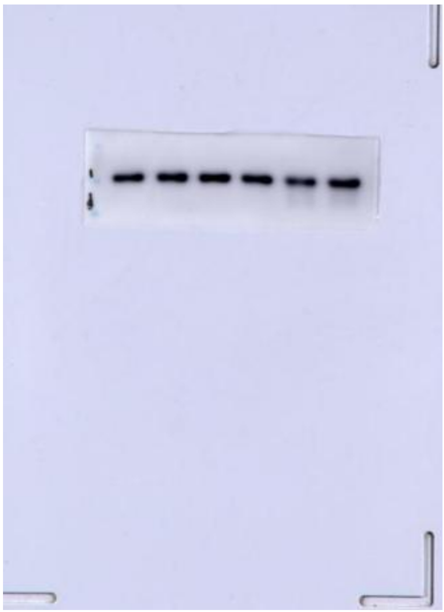

β-actin

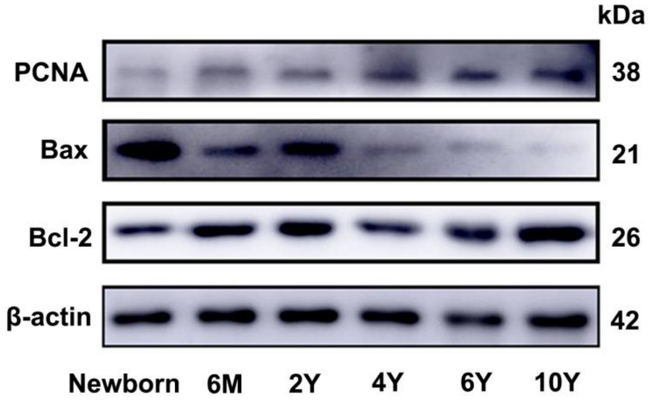
kDa
PCNA
38
Bax
21
Bcl-2
26
β-actin
42
Newborn
6M
2Y
4Y
6Y
10Y

Figure 2C

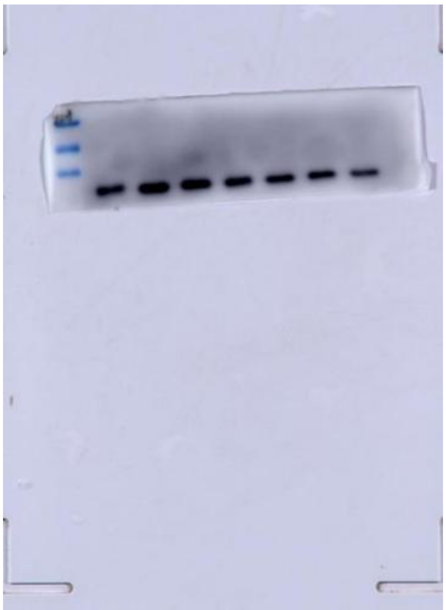

Bax

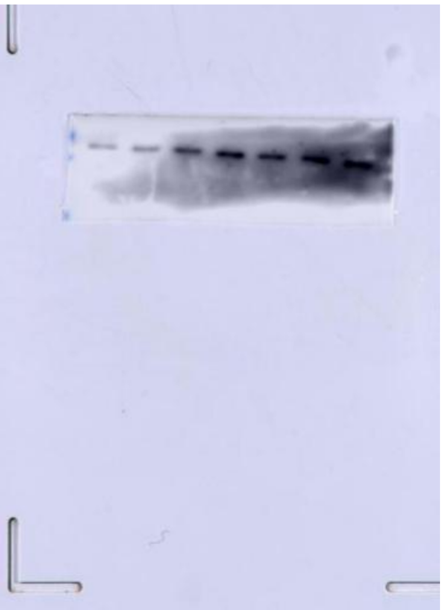

Bcl-2

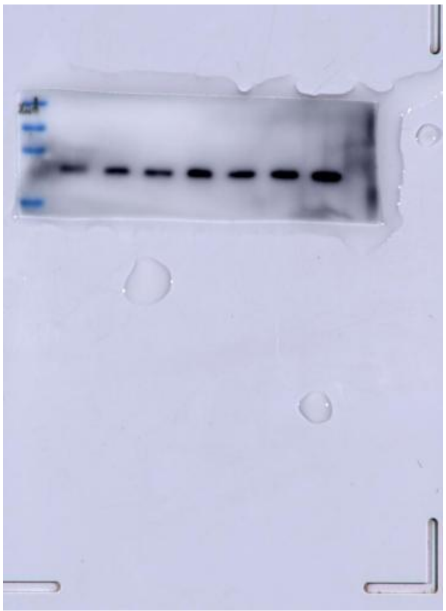

PCNA

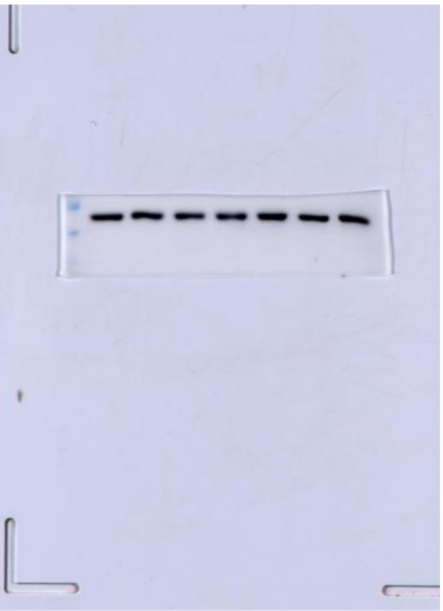

β-actin

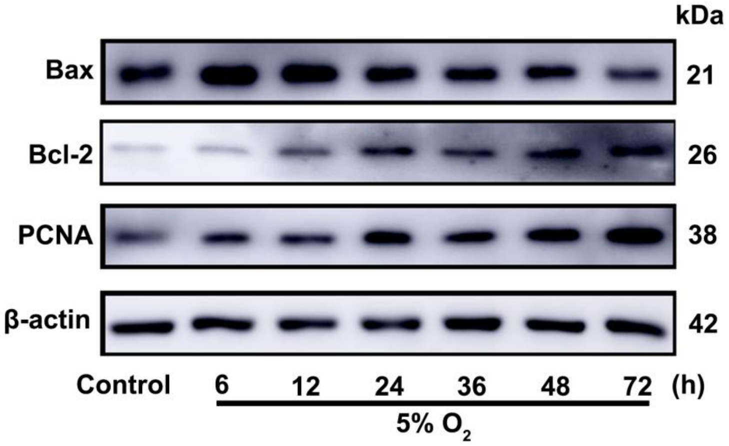
kDa
Bax
21
Bcl-2
26
PCNA
38
β-actin
42
Control
6
12
24
36
48
72
(h)
5% O2

Figure 3A

12 h

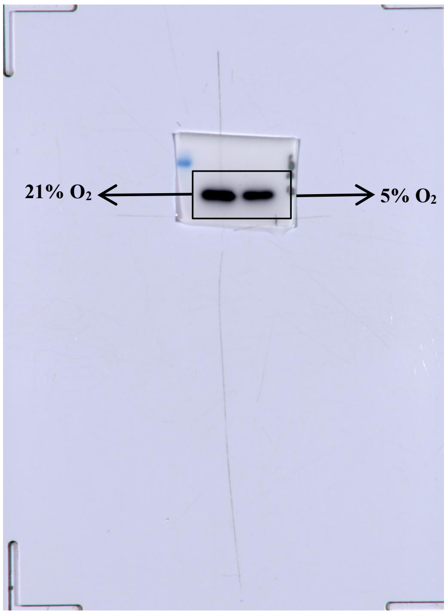

Bax

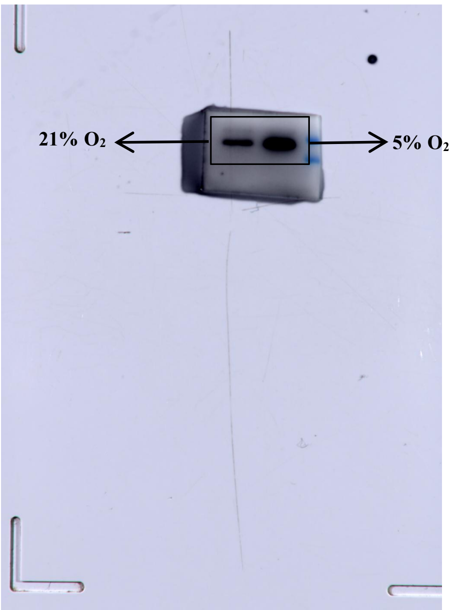

Bcl-2

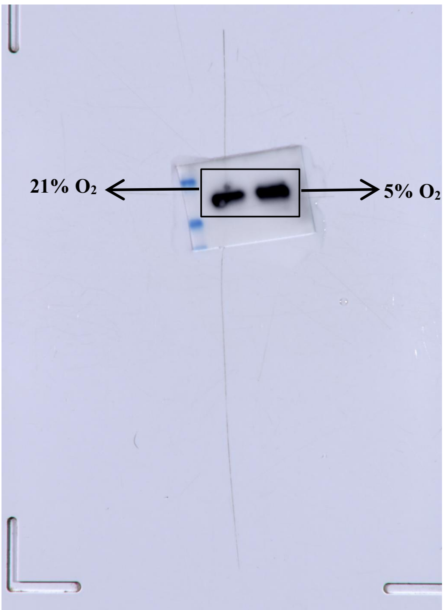

PCNA

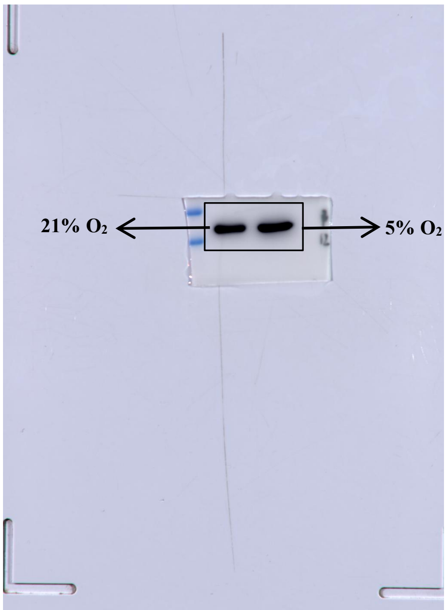

β-actin

24 h

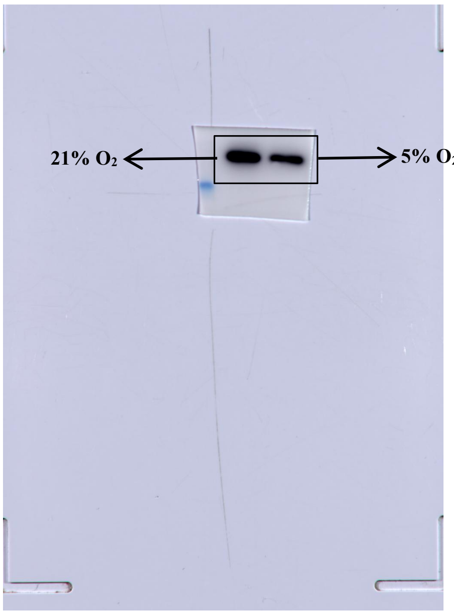

Bax

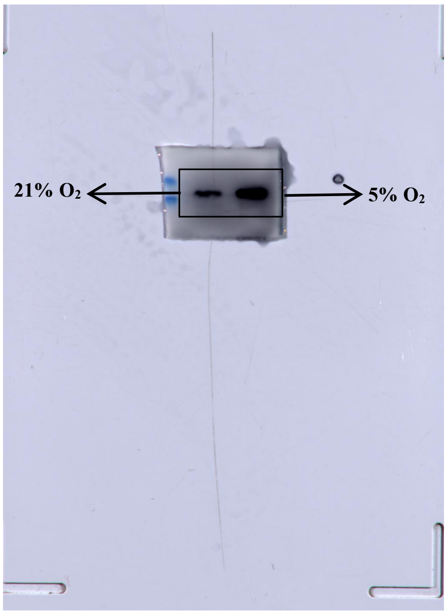

Bcl-2

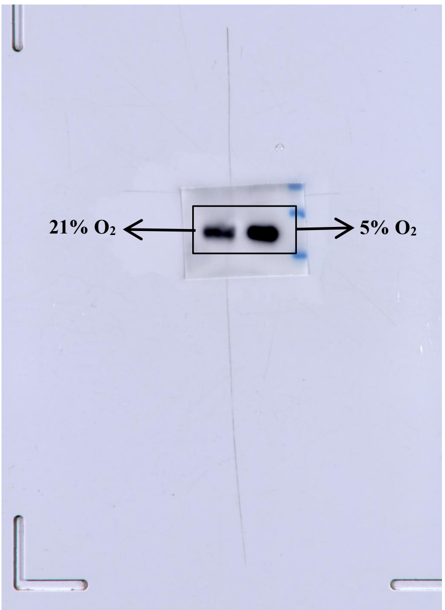

PCNA

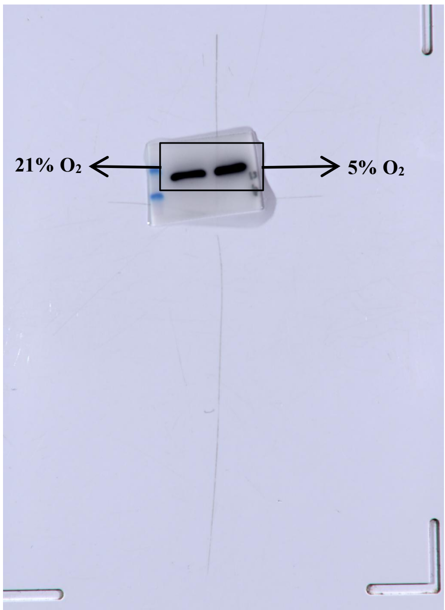

β-actin

48 h

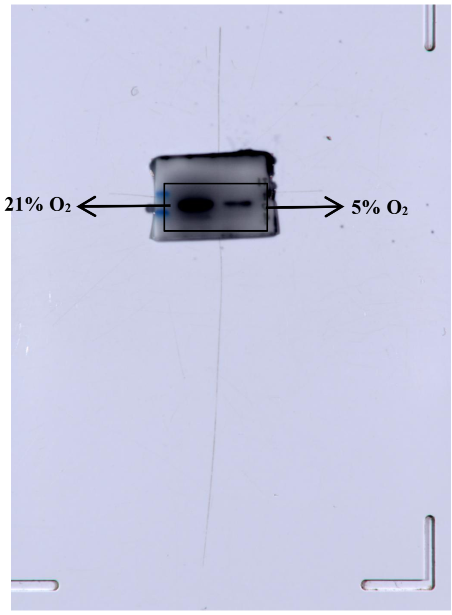

Bax

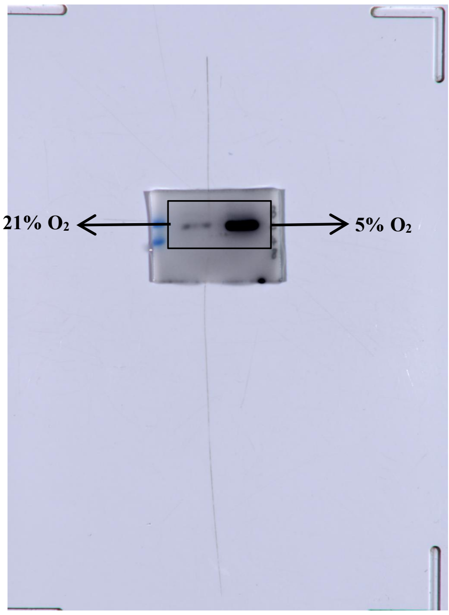

Bcl-2

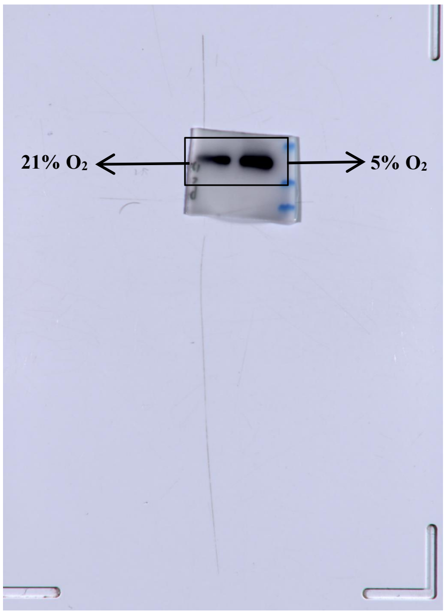

PCNA

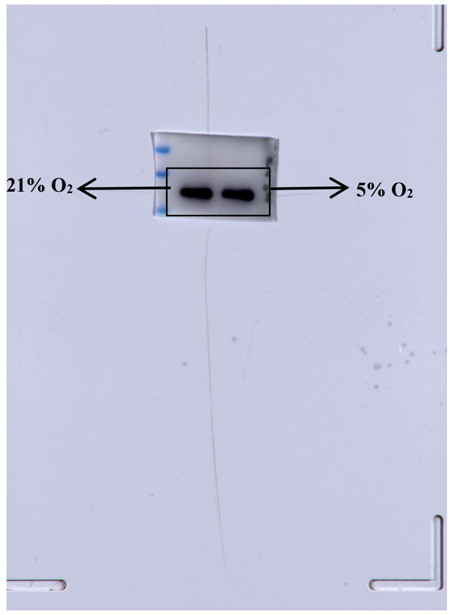

β-actin

72 h

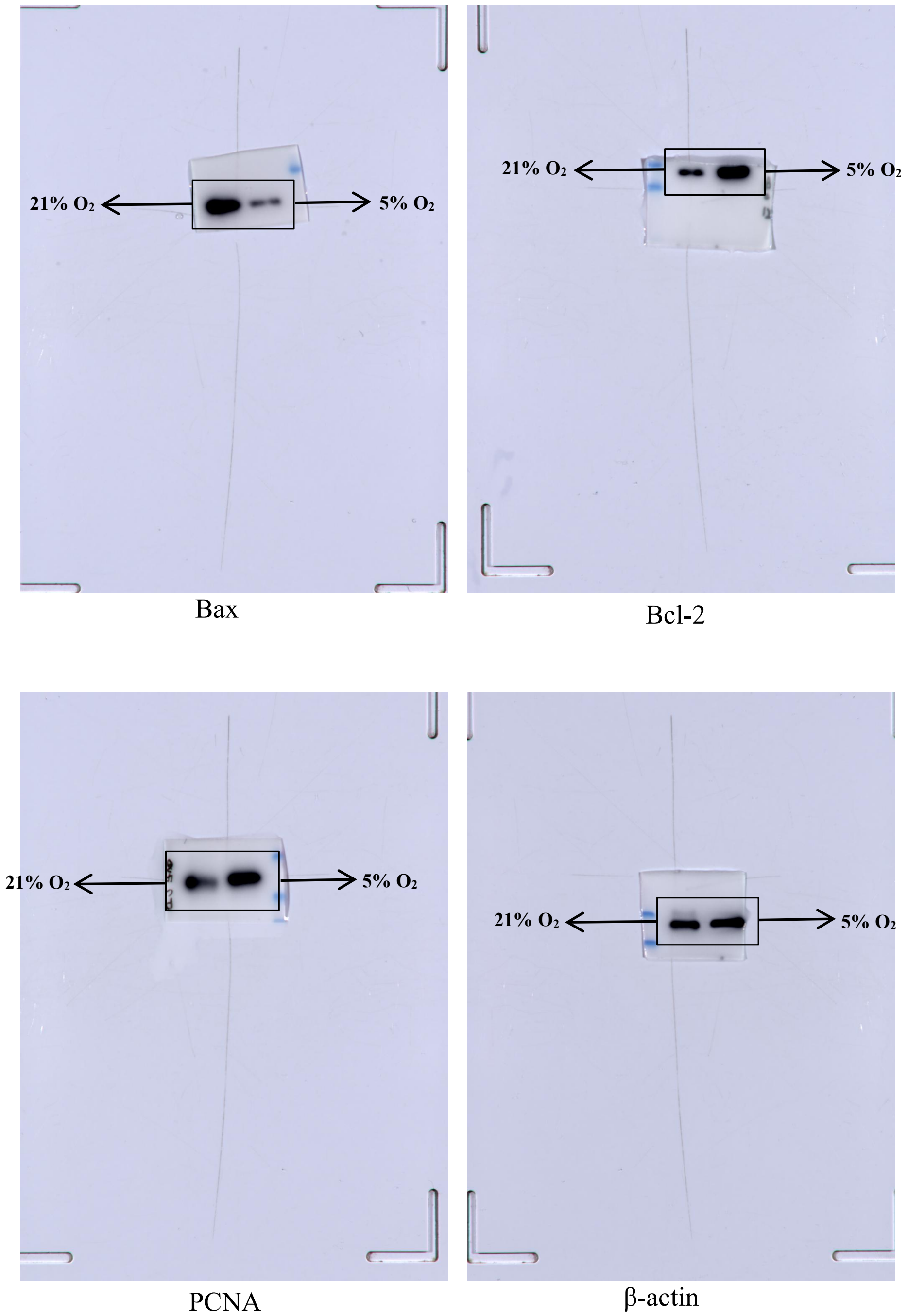

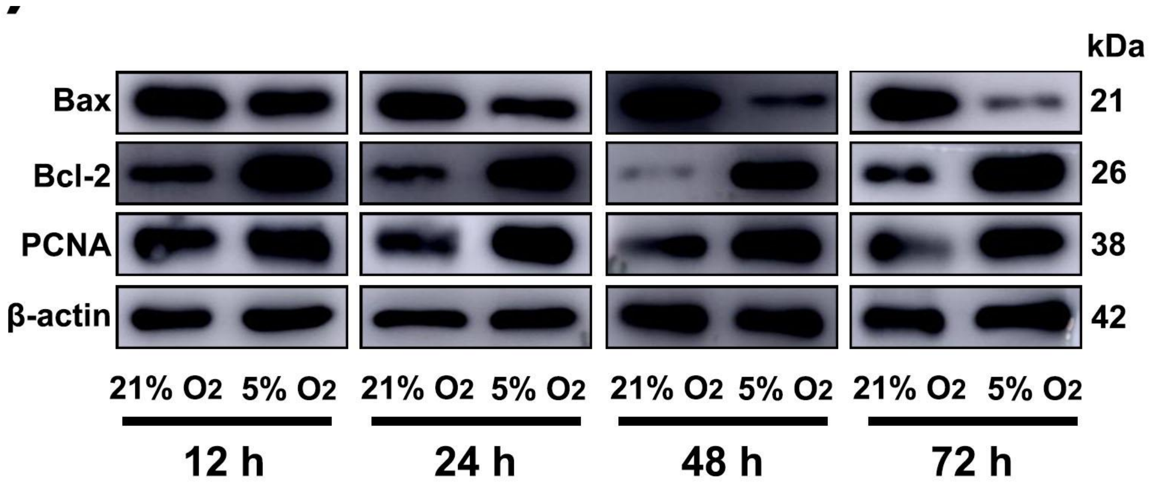

kDa
Bax
Bcl-2
PCNA
β-actin
21
26
38
42
21% O2
5% O2
21% O2
5% O2
21% O2
5% O2
21% O2
5% O2
12 h
24 h
48 h
72 h

Figure 4B, 4E

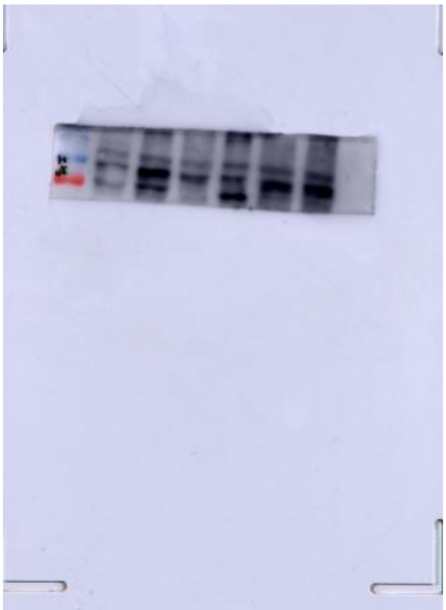

HIF-1α

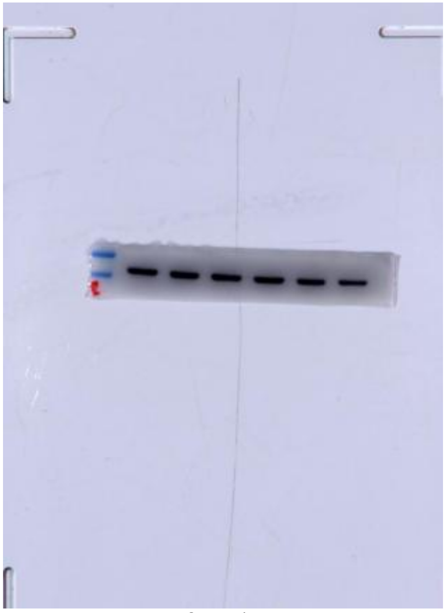

β-actin

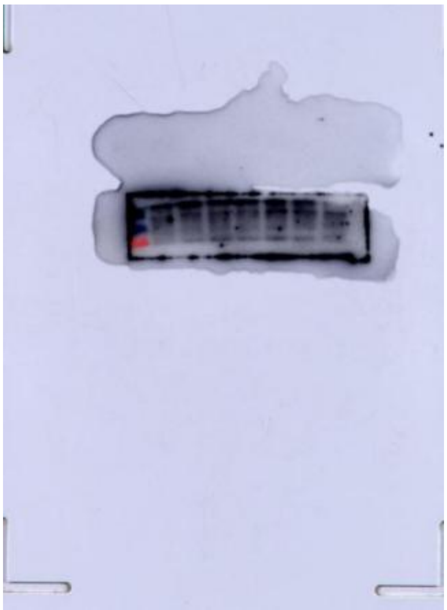

HIF-1α

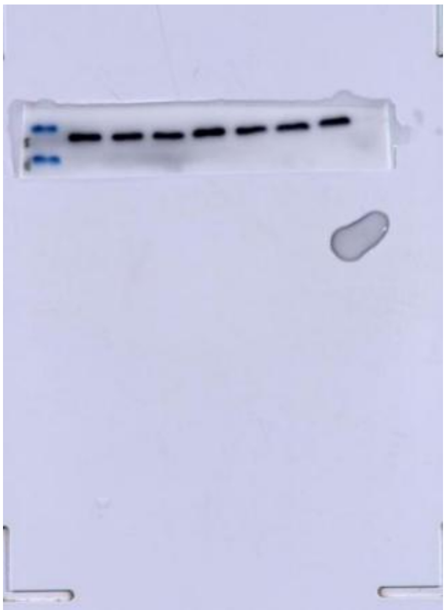

β-actin

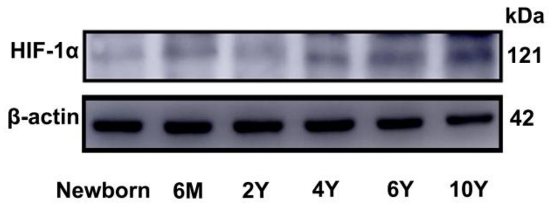
kDa
HIF-1α
121
β-actin
42
Newborn
6M
2Y
4Y
6Y
10Y

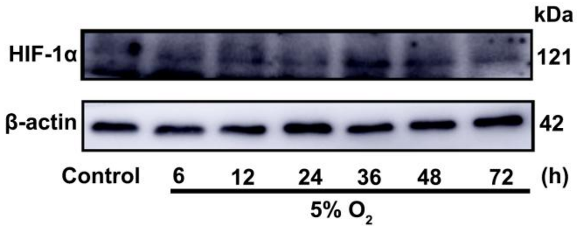
kDa
HIF-1α
121
β-actin
42
Control
6
12
24
36
48
72
(h)
5% O2

Figure 5A

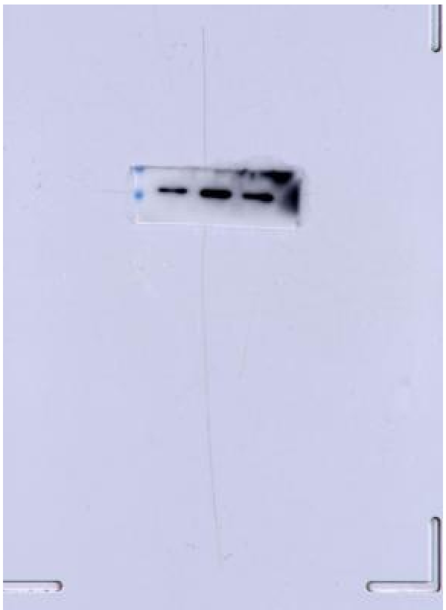

PCNA（DMOG）

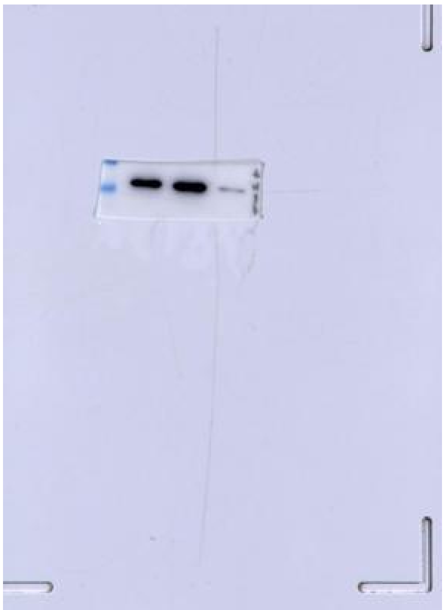

PCNA（LW6）

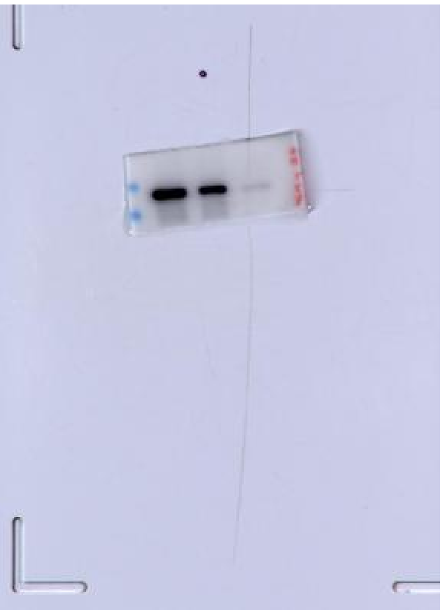

Bax（DMOG）

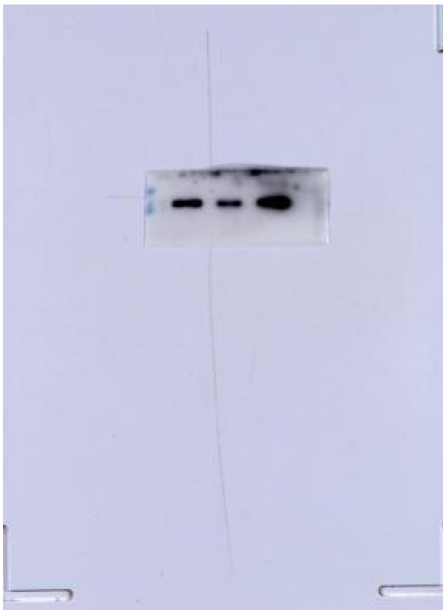

Bax（LW6）

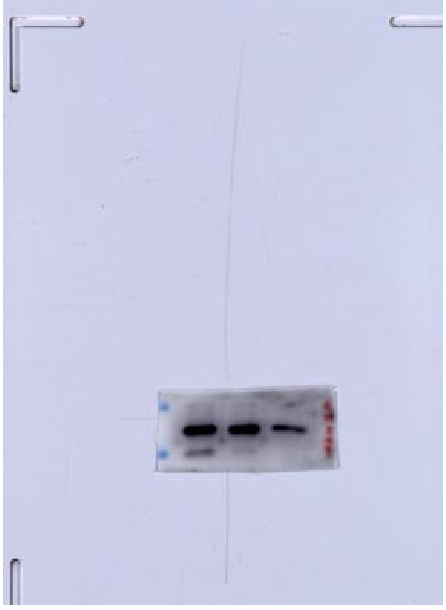
Bcl-2（DMOG）

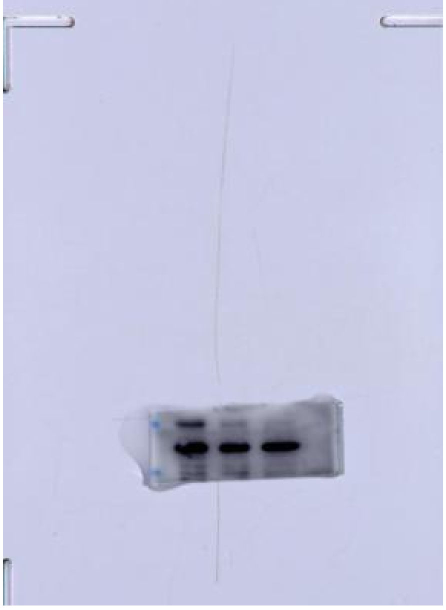
Bcl-2（LW6）

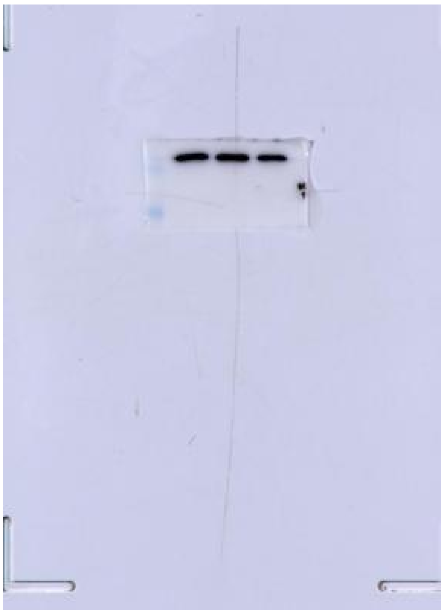
β-actin（DMOG）

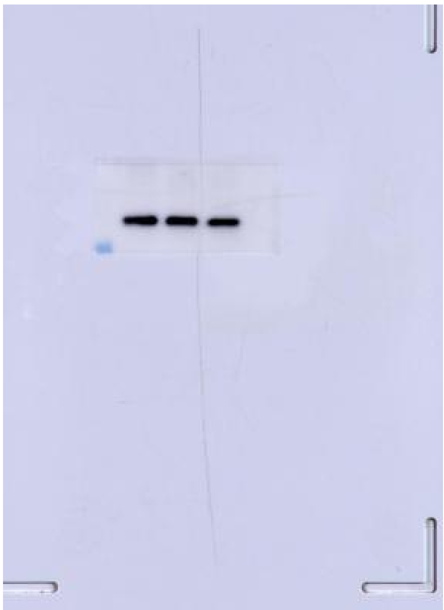
β-actin（LW6）

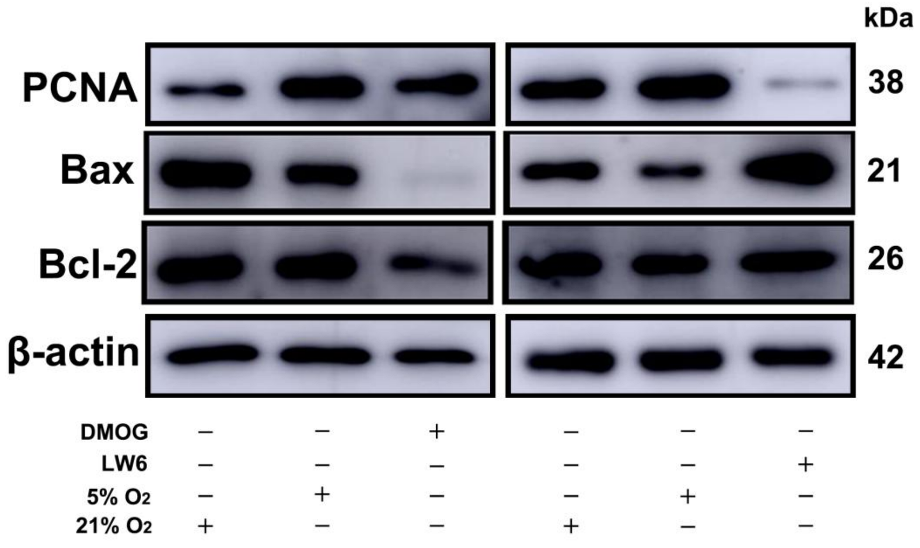
kDa
PCNA
38
Bax
21
Bcl-2
26
β-actin
42
DMOG – – + – – –
LW6 – – – – – +
5% O2 – + – – + –
21% O2 + – – + – –

Figure 6A

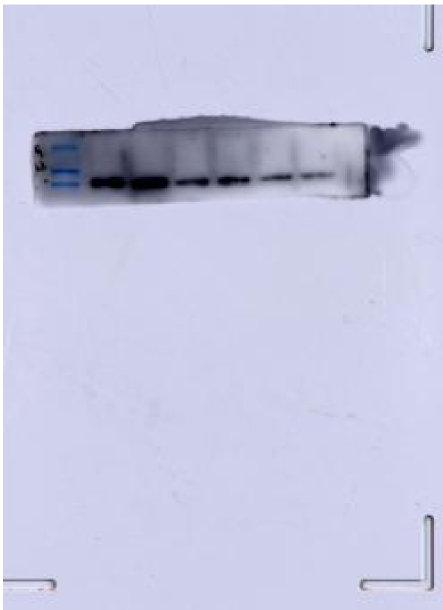

P62

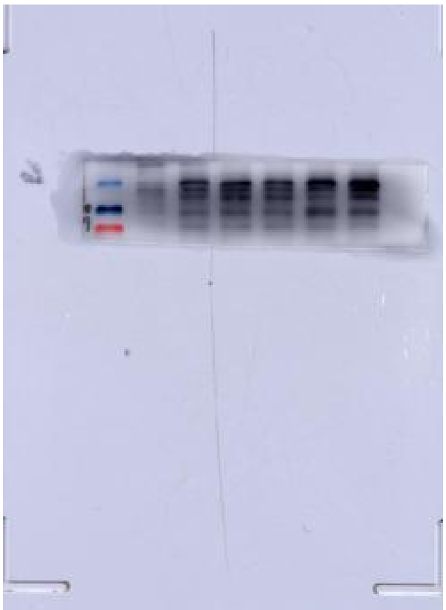

Beclin1

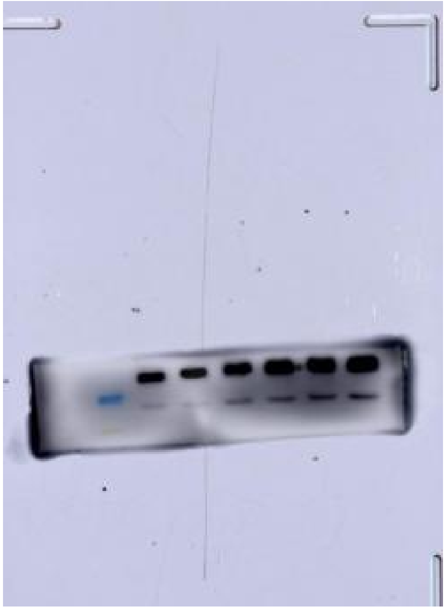

LC3

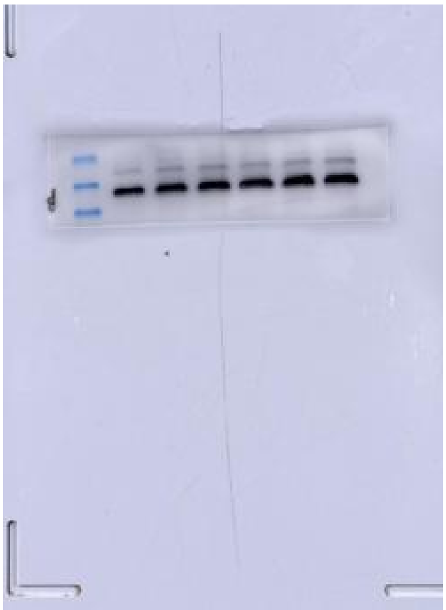

β-actin

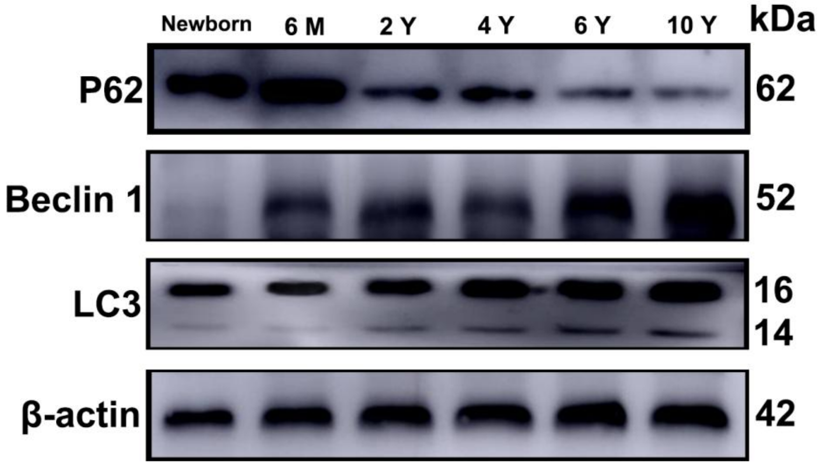
Newborn
6 M
2 Y
4 Y
6 Y
10 Y
kDa
P62
62
Beclin 1
52
LC3
16
14
β-actin
42

Figure 7A

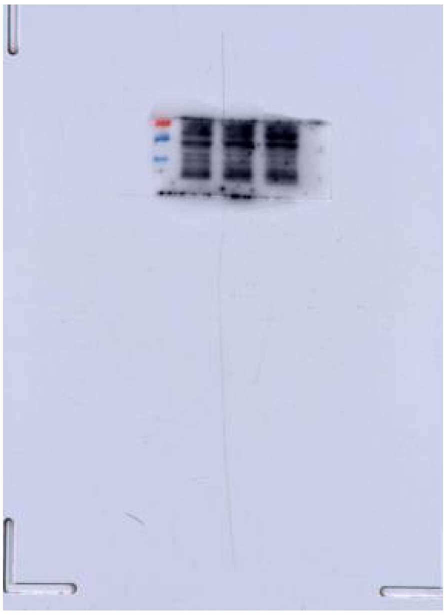

Beclin1（DMOG）

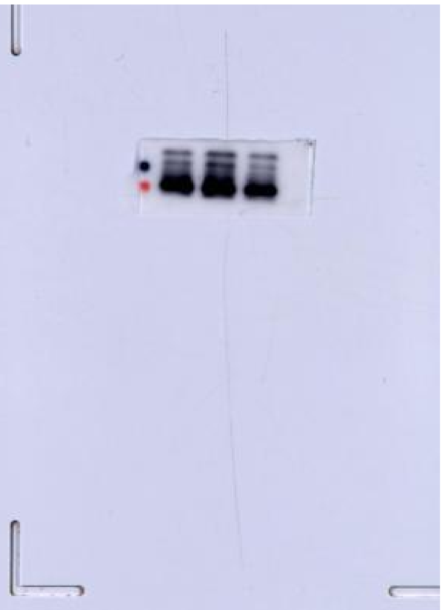

Beclin1（LW6）

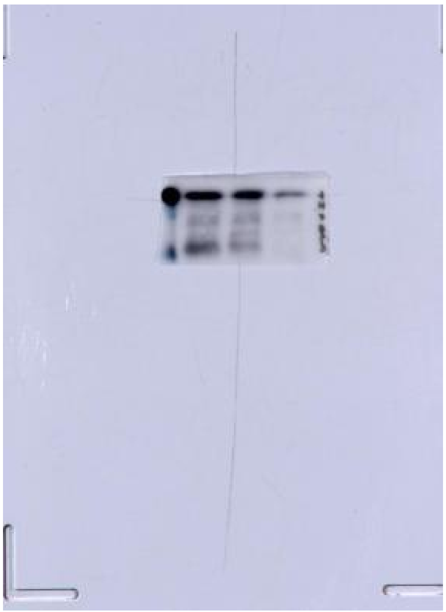

P62（DMOG）

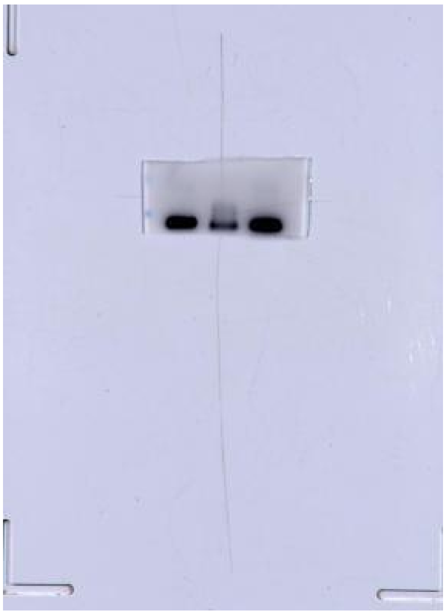

P62（LW6）

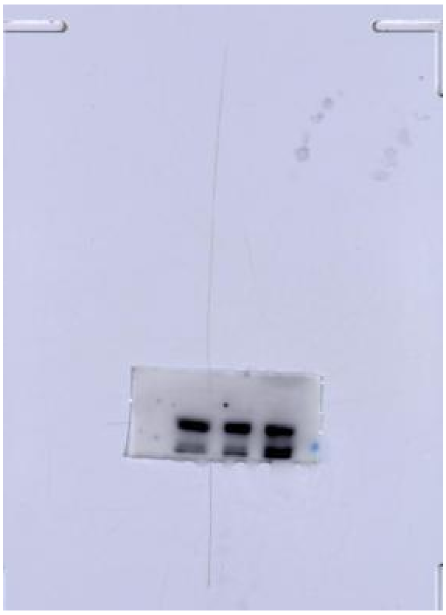

LC3（DMOG）

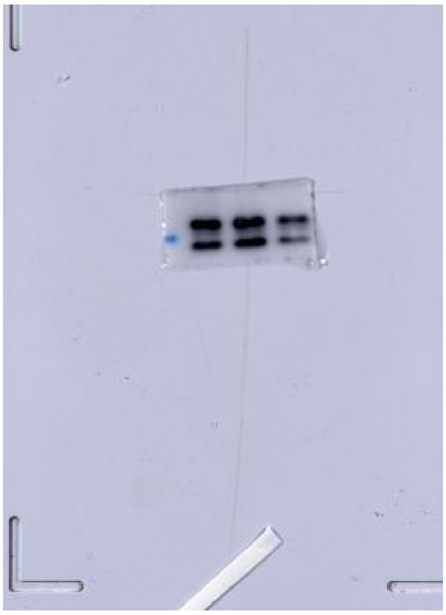

LC3（LW6）

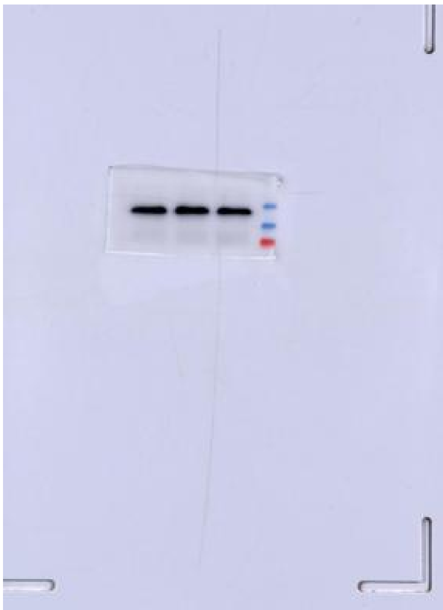

β-actin（DMOG）

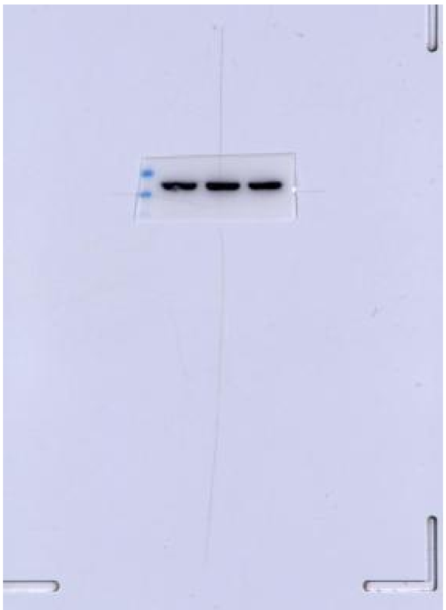

β-actin（LW6）

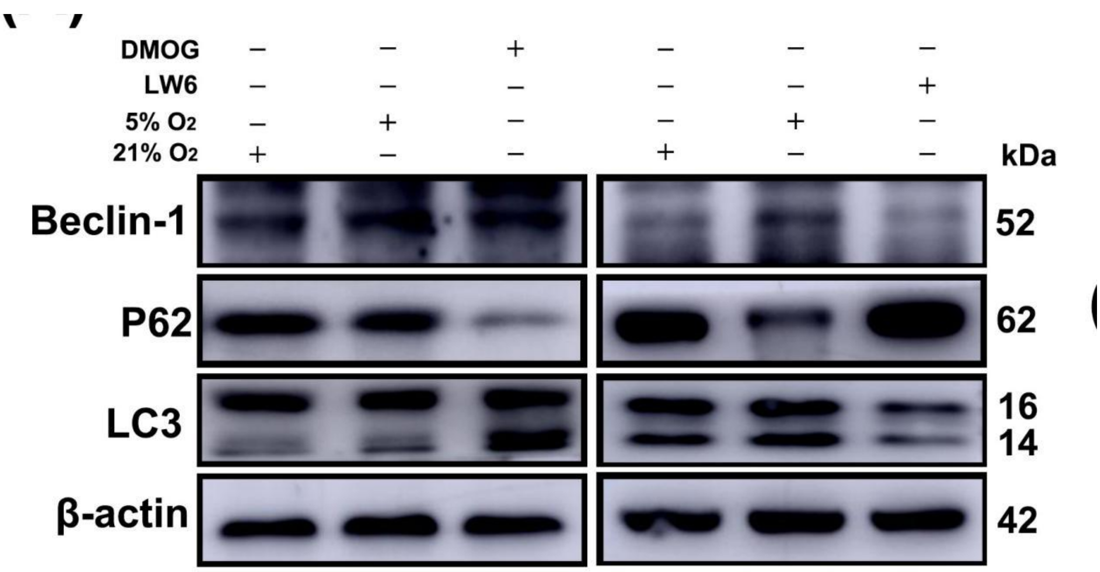
DMOG
LW6
5% O2
21% O2
kDa
Beclin-1
P62
LC3
β-actin
52
62
16
14
42

Figure 8A, 8G

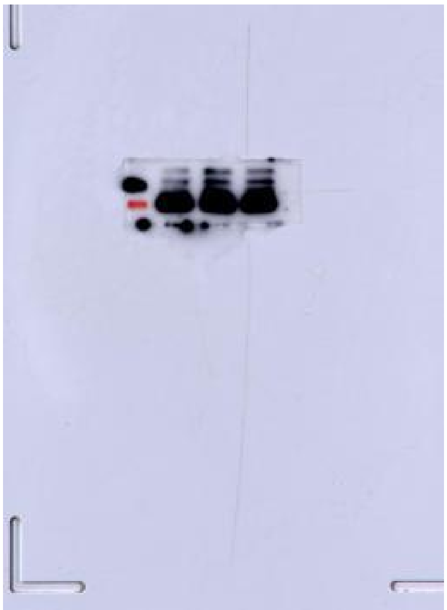

Beclin1（RAPA）

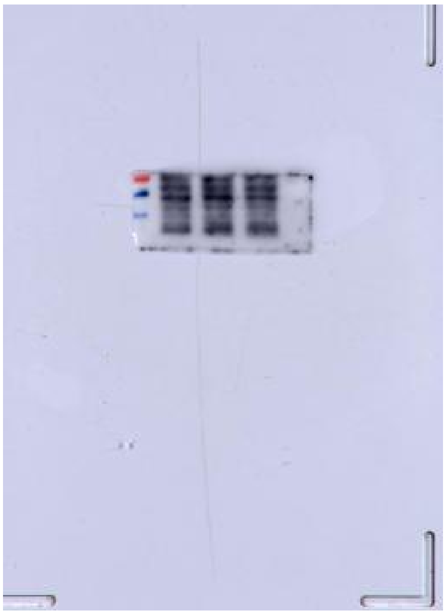

Beclin1（CQ）

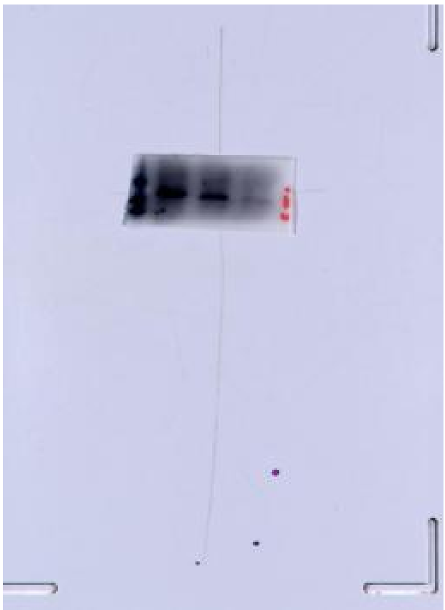

P62（RAPA）

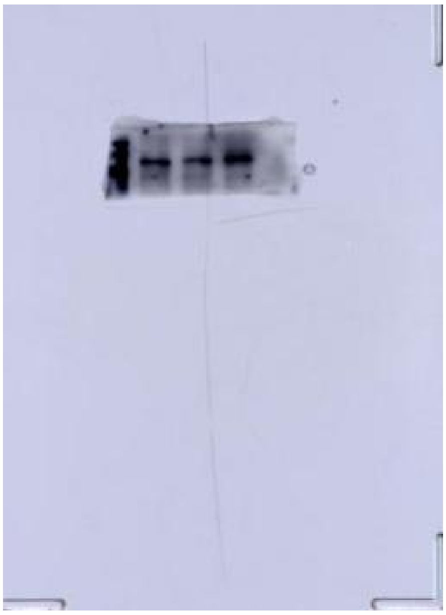

P62（CQ）

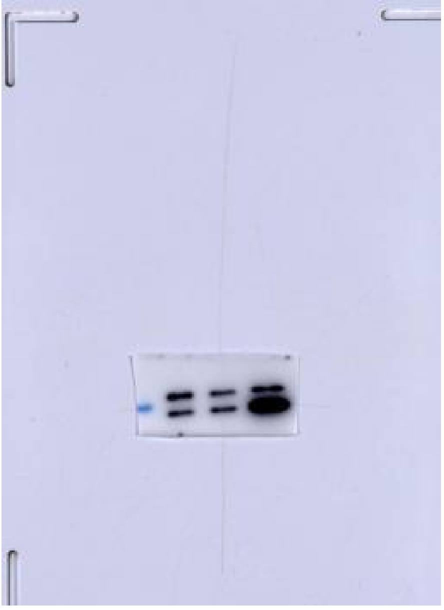

LC3（RAPA）

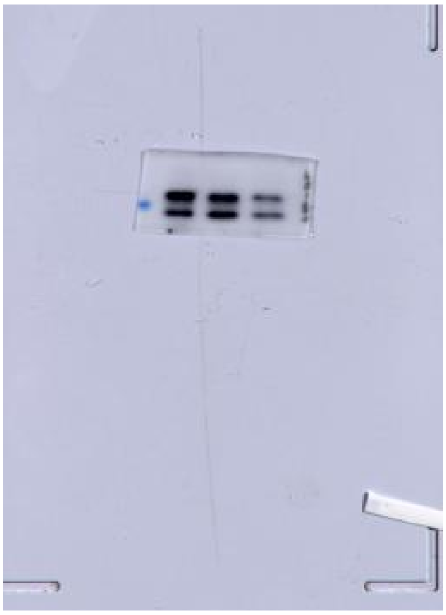

LC3（CQ）

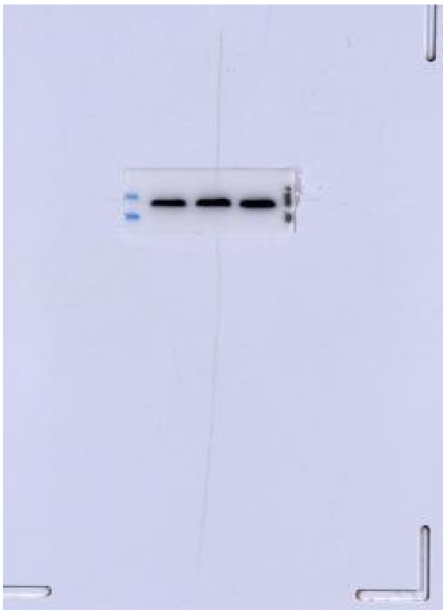

β-actin（RAPA）

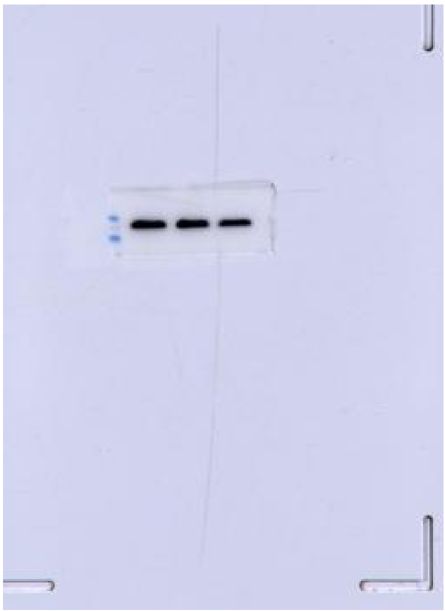

β-actin（CQ）

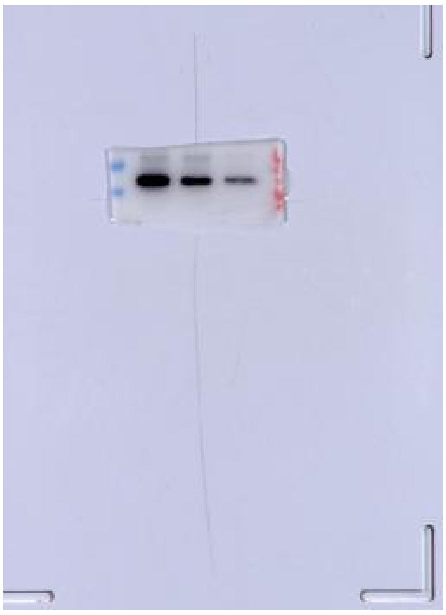

Bax（RAPA）

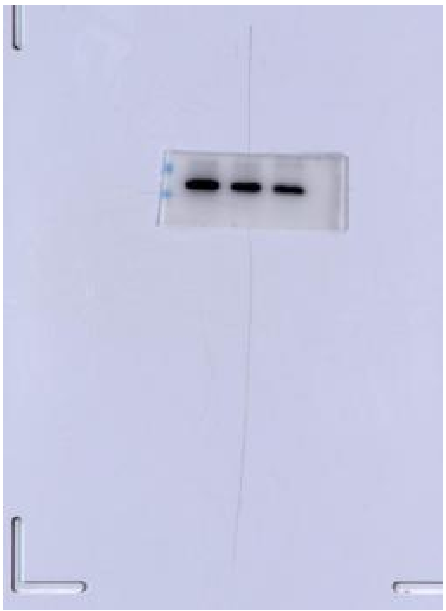

Bax（CQ）

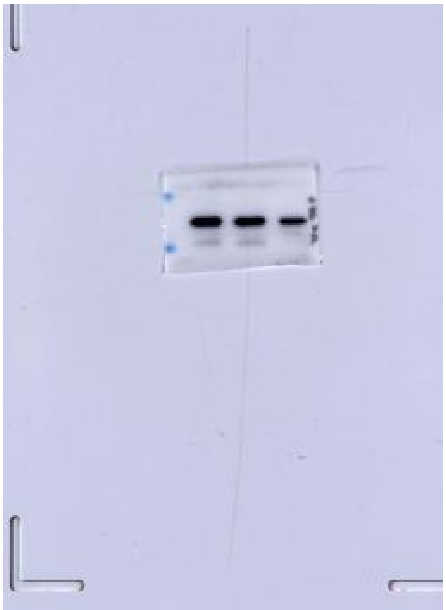

Bcl-2（RAPA）

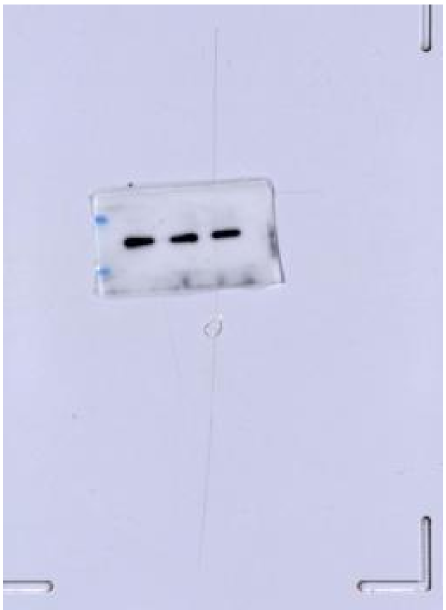

Bcl-2（CQ）

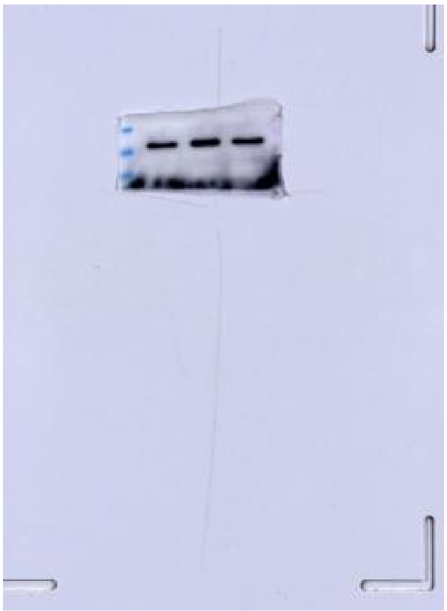

PCNA（RAPA）

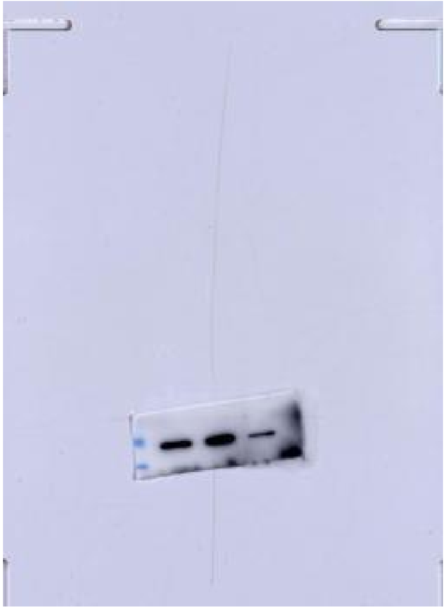

PCNA（CQ）

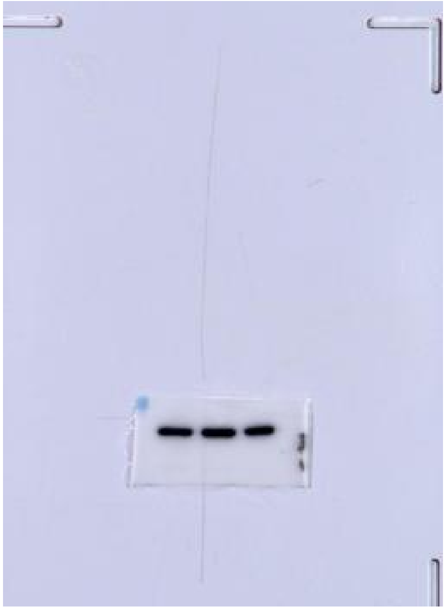

β-actin（RAPA）

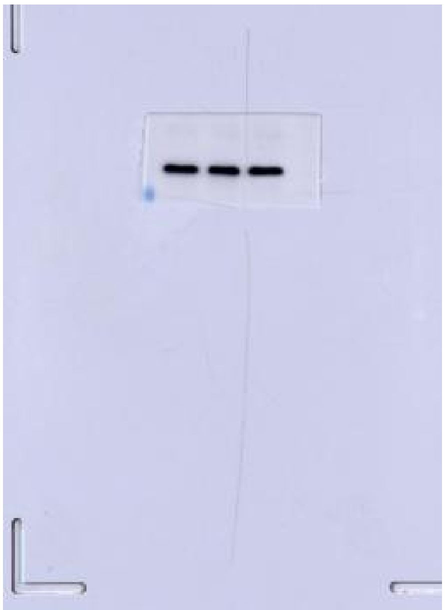

β-actin（CQ）

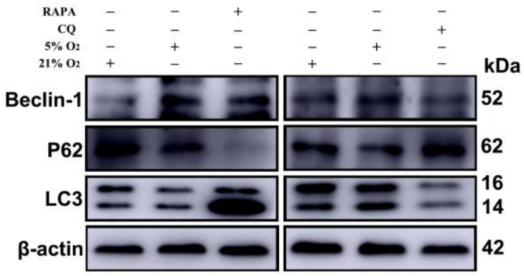

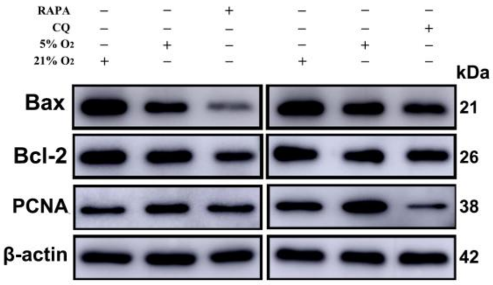

Supplement: Supplementary file 1 [file biomolecules-15-00256-s001.zip › biomolecules-3435144-supplementary.pdf]
